# Risk Factors for Postural Tachycardia Syndrome in Children and Adolescents

**DOI:** 10.1371/journal.pone.0113625

**Published:** 2014-12-04

**Authors:** Jing Lin, Zhenhui Han, Xueying Li, Todd Ochs, Juan Zhao, Xi Zhang, Jinyan Yang, Ping Liu, Zhenyu Xiong, Yong Gai, Chaoshu Tang, Junbao Du, Hongfang Jin

**Affiliations:** 1 Department of Pediatrics, Peking University First Hospital, Beijing, China; 2 Department of Pediatrics, Children's Hospital of Kaifeng, Henan, China; 3 Department of Medical Statistics, Peking University First Hospital, Beijing, China; 4 Department of Pediatrics, Northwestern Feinberg School of Medicine, Chicago, United States of America; 5 Department of Physiology and Pathophysiology, Peking University Health Sciences Centre, Beijing, China; 6 Key Laboratory of Molecular Cardiology, Ministry of Education, Beijing, China; National Taiwan University Hospital, Taiwan

## Abstract

**Background:**

Postural tachycardia syndrome (POTS) is prevalent in children and adolescents and has a great impact on health. But its risk factors have not been fully understood. This study aimed to explore possible risk factors for children and adolescents with POTS.

**Methods and Findings:**

600 children and adolescents (test group) aged 7–18 (11.9±3.0) years old, 259 males and 341 females, were recruited for identifying its risk factors. Another 197 subjects aged from 7 to 18 (11.3±2.3) years old were enrolled in the validation group. Heart rate (HR) and blood pressure (BP) were monitored during upright test. Risk factors were analyzed and sensitivity and specificity for predicting POTS were tested via receiver operating characteristic curve. Among 600 subjects, 41 were confirmed with POTS patients (6.8%) based on clinical manifestation and upright test. The results showed a significant difference in daily water intake, the daily sleeping hours, supine HR, HR increment and maximum HR during upright test between POTS and the unaffected children (*P*<0.05). Likelihood of POTS would increase by 1.583 times if supine HR was increased by 10 beats/min (95%CI 1.184 to 2.116, *P*<0.01), by 3.877 times if a child's water intake was less than 800 ml/day (95%CI 1.937 to 7.760, *P*<0.001), or by 5.905 times (95%CI 2.972 to 11.733, *P*<0.001) if sleeping hours were less than 8 hours/day. Supine HR, daily water intake and sleeping hours showed the capability of predicting POTS in children and adolescents with an AUC of 83.9% (95% CI: 78.6%–89.1%), sensitivity of 80.5% and specificity of 75%. Furthermore, in validation group, predictive sensitivity and specificity were 73.3% and 72.5%.

**Conclusion:**

Faster supine HR, less water intake and shorter sleeping hours were identified as risk factors for POTS.

## Introduction

Postural tachycardia syndrome (POTS) is a chronic condition with frequent symptoms of orthostatic intolerance or with sympathetic activation and excessive tachycardia while standing, without significant hypotension [1], [2]. Orthostatic symptoms include dizziness, lightheadedness, near syncope, weakness in legs, blurred vision or transient “blackout” or “whiteout” of vision, headache, neck pain, nausea, poor concentration, and occasional syncope. Symptoms of sympathetic activation include palpitations, chest pain, vasomotor skin changes, warm feeling, and tremulousness [1]. POTS is a multi-system condition with heterogeneous clinical features and pathophysiology that can be quite disabling, and deleterious with significant effects on individual quality of life [3]–[8].

Up to now, there have been some data concerning the prevalence of POTS in adults and young males. Low et al. reported that the incidence of POTS was 170/100000 in adults [9]. A study showed that the prevalence of POTS in young males around 10% [10]. However, the data of the current prevalence of POTS in children and adolescents at a wider range of age is lacking.

For the purpose of the prevention of such disease, elucidating the risk factors of POTS in children is crucial to develop effective preventive strategies. Studies indicated that POTS patients, with hypovolemia, had inadequate venous return to the heart in an upright position [11]–[13]. Patients with POTS had decreased urinary sodium content in a 24-hour collection, when compared to normal subjects. After saline loading, the affected children's urinary sodium increased, and their clinical symptoms improved [14]. This supported the premise that fluid intake and blood volume were directly related to the development of POTS. However, it is unclear if reduced fluid intake would also be a risk factor for POTS. Previous studies have suggested that compared with normal subjects, children with POTS sleep less. With fatigue, the symptoms of POTS get worse [15], [16]. These results suggest that inadequate sleep might be related to the pathophysiology of POTS. Up until now, however, evidence has been lacking.

Thus, the present study was undertaken to investigate the prevalence rate of POTS in children and adolescents in China, and identify the risk factors for this disease.

## Subjects and Methods

This study was approved by the Ethics Committee in Peking University First Hospital. Written informed consent was obtained from all study subjects/parents, the next of kin, caretakers, or guardians of the minors/children enrolled in our study.

### Subjects

A primary school, a middle school, and a high school were randomly selected in Kaifeng city, Henan province, in China. A total of 600 children and adolescents, drawn from the first two classes of each grade, were enrolled in this study as a test group, to ascertain POTS risk factors. Their age ranged from 7 to 18 (11.9±3.0) years old. Males numbered 259 (43.2%) and females numbered 341 (56.8%). Another group of 197 children, who were randomly selected from those schools served as a validation group, including 115 males and 82 females, ranging in age from 7–18 (11.3±2.3) years old. Those who had a history of surgery, pregnancy, chronic diseases, infectious diseases, autoimmune diseases and psychomotor developmental disabilities were excluded. The underlying causes that may either secondarily cause POTS or co-occur with POTS, such as anemia, dehydration, or hyperthyroidism were excluded by detailed history taking, physical examination and laboratory tests when necessary. Those who were taking drugs affecting autonomic function were excluded. All the children who were diagnosed as POTS repeated examinations to make sure there were no possible underlying diseases. For children and adolescents who met the criteria of a positive response, the examiners further examined the children again by repeating the medical history review and upright test to make sure he or she had the “chronic condition” with chronic symptoms of orthostatic intolerance for last at least 3 months. Written informed consent was obtained from all study subjects/parents, the next of kin, caretakers, or guardians of the minors/children enrolled in our study.

### Questionnaire

The questionnaire was completed with the investigators interviewing the participants, in-person. The content of the questionnaire included the name, gender, date of birth, home address, telephone number and other basic information, as well as history of syncope, history of car sickness, syncope in the family history, water intake, sleeping hours, as well as information about the school-induced stress. We asked subjects to provide the information about the daily water intake with the help of parents, referring to the cups with scale for the purpose of understanding the amount of daily water intake and it was defined in the questionnaire with the reference of previous studies [17], [18]. The sleeping hours in our questionnaire were defined as an average of the sleeping hrs in a week. We asked subjects to provide the information about the sleeping hours within a week with the help of parents.

### Height and Weight Measurements

The height and weight of each participant were measured with a Height and Weight Tester HW-600 (Kaiyuan Electronics Co., Ltd., Zhengzhou, China). Before the measurement, participants removed their shoes, socks and hats. Each child stood upright on the scale platform with his/her back against the height measuring rod and with eyes looking straight ahead. Then, the examiner adjusted the measuring plate, until it contacted with the child's head. Height was recorded to the nearest centimeter. The weight and height were measured simultaneously. The children avoided exercise and urinated, prior to taking the measurements. Weight was recorded to the nearest kilogram.

### Upright Test

Participants remained supine on the bed for at least ten min, in a room with quiet environment and a suitable temperature. Then, they were asked to stand. During the ten min in an upright position, their blood pressure (BP) was monitored and their heart rate (HR) was recorded. HR and BP in both the supine and upright positions were monitored with Dash 2000 multi-lead ECG (General Electric, NY, New York, USA). In the upright test, we derived 4 BPs for each subject, 1 supine BP and 3 standing BPs at 3 minutes, 6 minutes and 9 minutes, respectively, after standing. The criteria for a positive reaction (POTS) were as follows: the HR increased by over 40 beats/min or the maximum HR was over 120 beats/min, and the patients had at least one of the following symptoms during the ten minutes standing: dizziness or fainting, chest tightness, headache, palpitations, blurred vision, fatigue or syncope for at least 3 months [11], [19]–[22].

### Quality Assurance

Before the study began, the investigators were professionally trained, and they were allowed to participate in this study only after a written assessment. Two technical supervisors were placed in charge of the investigational work. After the questionnaires were administered, 5%–10% of them were randomly checked.

### Statistical analysis

All the data were placed into a prepared Excel table and SPSS17.0 software was used to perform the t-test, Chi-square test. Multivariate Logistic regression analysis was used, to which the variables contributed significantly to the incidence of POTS were analyzed, then yielding the odds ratios. The receiver operating characteristic curve (ROC) was used to assess the sensitivity and specificity of using the study factors in predicting POTS. The area under curve (AUC) indicated the predictive value of the study factors. An AUC from 0.5 to 0.7 means a low predictive value; AUC from 0.7 to 0.9 means a moderate predictive value; and AUC>0.9 means a high predictive value. Optimal cutoff value was determined by the maximum of Youden index, which is defined as sensitivity plus specificity minus 1, where sensitivity and specificity were calculated as proportions. To validate the methodology, and verify the chosen risk factors for POTS in the general population, another 197 children were examined, using the study protocol.

### Ethical standards

The authors asserted that all procedures contributing to this work complied with the ethical standards of the relevant national guidelines on human experimentation and with the Helsinki Declaration on 1975, as revised in 2008, and approved by Peking University First Hospital Ethics Committee. All procedures contributing to this work comply with the ethical standards expressed in relevant national guidelines.

We obtained the written informed consent from all the parents of the participants, the next of kin, caretakers, or guardians of the minors/children enrolled in our study. We recorded participant written consent well. This was approved by Peking University First Hospital Ethics Committee.

## Results

### Prevalence of POTS in children and adolescents

Of the 600 cases enrolled as the test group, the participants had an average age of 11.9±3.0 years old, ranging from 7 to 18 years old. Boys constituted 43.2% of the sample (n = 259) and girls constituted 56.8% (n = 341). Forty-one children met the diagnostic standard for POTS, constituting for 6.8% of the test group. In the 41 patients, males were 16, accounting for 2.7%, and females 25, accounting for 4.2%. No statistically significant differences in the prevalence rates of POTS were found between males and females (*p*>0.05).

### The participant characteristics between normal and the POTS in children and adolescents

According to the diagnostic criteria for POTS, the distribution of participant characteristics between normal and the POTS children are listed in Table 1. There were significant differences between the POTS children and the normal children in daily water intake, and daily hours of sleep (*P*<0.05). Also, there were significant differences between the two groups in supine HR, HR increment and their maximum HR within the first 10 min after standing (*P*<0.05).

**Table 1:** Distribution of characteristics in participants of test group.

Characteristics	Normal subjects	POTS subjects	χ2/t	*p* Value
Cases, n	559	41	-	-
Male/female	243/316	16/25	0.308	0.579
Age, yrs	11.9±3.0	12.1±2.5	−0.503	0.617
BMI (kg/m^2^)	19.0±3.7	18.7±3.0	0.601	0.548
Supine SBP, mmHg	110±10	110±10	−0.029	0.977
Supine DBP, mmHg	65±9	65±8	−0.166	0.868
Supine HR, beats/min	84±11	89±12	−2.419	0.020
Upright SBP, mmHg	118±10	120±11	−1.111	0.267
Upright DBP, mmHg	76±9	77±8	−0.591	0.554
Upright highest HR, beats/min	106±12	124±6	−16.062	<0.001
HR increment, beats/min	22±8	35±10	−8.480	<0.001
Water intake (less/more)	148/411	25/16	22.157	<0.001
Sleeping hours (<8 h/≥8 h)	88/471	23/18	41.258	<0.001
School-induced stress (more/less)	317/242	26/15	0.702	0.402
Car-sickness (yes/no)	302/257	26/15	1.359	0.244
Family history (yes/no)	99/460	10/31	1.147	0.284

POTS: postural orthostatic tachycardia syndrome; HR: Heart rate; SBP: Systolic blood pressure; DBP: Diastolic blood pressure.

### Risk factors for POTS in children and adolescents

Using Logistic analysis, the supine HR, hours of sleep, and water intake were identified as the three risk factors, which contributed to the prediction of POTS. If the supine HR was faster by 10 beats/min, the risk of suffering from POTS was increased 1.583 times (95%CI 1.184 to 2.116, *P*<0.001). If a child's water intake was less than 800 ml/day or his/her sleeping hours were less than 8 hours/day, POTS would be possibly present with the risk of 3.877 times (95%CI 1.937 to 7.760, *P*<0.001) and 5.905 times (95%CI 2.972 to 11.733, *P*<0.001), respectively, when compared with those who had a water intake of more than 800 ml/day or sleeping hours longer than 8 hours/day (Table 2).

**Table 2:** Logistic multivariate regression analysis of variables.

Characteristics	B	SE	Wald	*P*	OR (95%CI)
Supine HR/10	0.459	0.148	9.603	<0.01	1.583 (1.184–2.116)
Water intake	1.355	0.354	14.643	<0.001	3.877 (1.937–7.760)
Sleeping hours	1.776	0.350	25.691	<0.001	5.905 (2.972–11.733)
Constant	−7.761	1.378	31.710	<0.001	-

Supine HR/10: Supine heart rate was divided by 10. Characteristics enrolled in the Logistic multivariate regression analysis: gender, age, BMI, supine HR/10, supine SBP, car sick, family history, water intake, sleeping hours and school-induced stress.

The Logistic equation was as follows: Log (P) = −7.761+0.459×X_1_+1.355×X_2_+1.776×X_3_, where X_1_ stands for supine HR/10, X_2_ stands for daily water intake, and X_3_ stands for daily sleeping hours.

### Predictive capability of the factors for POTS in children and adolescents

**Figure 1:**
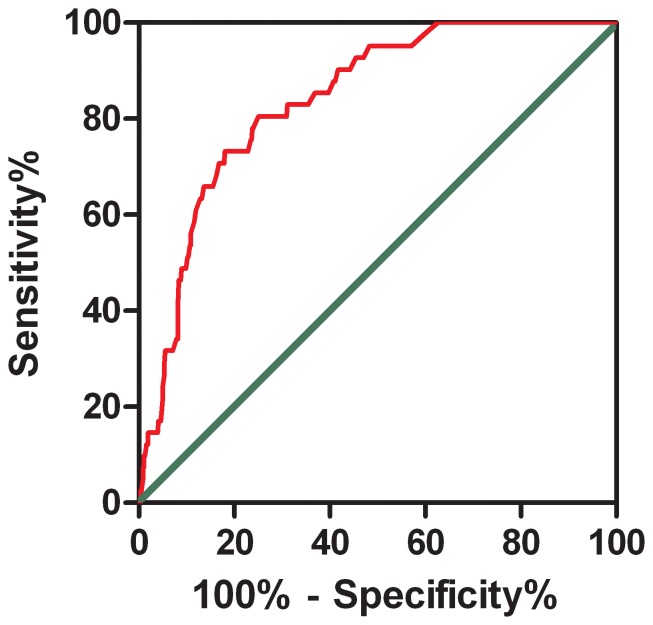
The receiver-operating characteristic (ROC) curve of the Logistic multivariate regression equation for predicting the likelihood of POTS. The longitudinal axis represents sensitivity to predict the probability of POTS. The transversal axis represents the false positive rate (1-specificity) of the prediction. The 45° gray line of the graph stands for reference line, representing sensitivity being equal to false positive rate (e.g., does not have the predictive value completely). The red curve is farther from the reference line and nearer the upper left corner of the graph. Area under the curve was 0.839 (95% confidence interval: 0.786 to 0.891; p<0.001).

To determine whether the three aforementioned factors could be regarded as true predictors for POTS in children, we applied ROC analysis. It showed that when the Youden Index was the highest, the cutoff point was −2.65, meaning that we could confidently predict POTS in a patient if the abovementioned result was equal to or greater than −2.65. Accordingly, the area under curve (AUC) was 83.9%, the sensitivity 80.5% and the specificity 75% (Figure 1).

### External validation

On the basis of the above findings, for the purpose of external confirmation, another 197 children and adolescents were enrolled as the validation group. The distribution of the participant characteristics between the normal children and the POTS children in the 197 participants are listed in Table 3. There were significant differences between the two groups in daily water intake, daily sleeping hours, supine DBP, HR increment and maximum HR within the first 10 min after standing (*P*<0.05).

**Table 3:** Distribution of characteristics of subjects in validation group.

Characteristics	Normal subjects	POTS subjects	χ2/t	*p* Value
Cases, n	182	15		
Male/female	103/79	12/3	3.125	0.077
Age, yrs	11.3±2.4	10.3±1.8	1.600	0.111
BMI (kg/m^2^)	18.1±3.2	18.5±3.3	−0.411	0.682
Supine SBP, mmHg	110±10	114±13	−1.088	0.293
Supine DBP, mmHg	64±10	71±10	−2.473	0.014
Supine HR, beats/min	86±11	94±16	−1.964	0.068
Upright SBP, mmHg	118±11	121±12	−0.966	0.335
Upright DBP, mmHg	77±10	81±12	−1.678	0.095
Upright highest HR, beats/min	107±11	126±10	−6.536	<0.001
HR increment, beats/min	22±7	32±12	−3.321	<0.01
Water intake (less/more)	41/141	11/4	18.411	<0.001
Sleeping hours (<8 h/≥8 h)	29/153	11/4	28.215	<0.001
School-induced stress (more/less)	96/86	10/5	1.080	0.299
Car-sickness (yes/no)	102/80	9/6	0.088	0.767
Family history (yes/no)	42/140	2/13	0.758	0.384

POTS: postural orthostatic tachycardia syndrome; HR: Heart rate; SBP: Systolic blood pressure; DBP: Diastolic blood pressure.

Then, we applied the diagnostic criteria to the validation group. The gold standard criteria for POTS were presented earlier. The proposed predictive paradigm included the three risk factors. The results showed that in the 197 children, 15 of them were diagnosed with POTS using the gold standard criteria, and 11 children were predicted correctly using the risk factors with a sensitivity of 73.3% and a specificity of 72.5% (Table 4).

**Table 4:** The diagnostic test of Logistic multivariate regression.

Logistic multivariate regression	Gold standard		
	+	−	Total
+	11	50	61
−	4	132	136
Total	15	182	197

## Discussion

Since POTS significantly impacts the health quality, great attention has been paid to its epidemic and prevention [23]–[25]. In recent years, researchers have reported an increasing number of children and youth with POTS. Stewart et al. proposed the concept of pediatric POTS in 1999 and codified it as a type of child orthostatic intolerance [26].

Through the present study we learned the prevalence rate of POTS in children under 18 years old. This was accomplished, using a cross-sectional investigation, performed in Kaifeng city, Henan province, China, where 600 Chinese children and adolescents were examined through questionnaires and the upright test. The prevalence rate of POTS in Chinese children and adolescents was 6.8%. It was reported that the prevalence rate in women was higher than that in men, with the prevalence rate of females being four times higher [27]. The results of the present study indicated that there was no significant difference in POTS prevalence between boys and girls.

In this investigation, we displayed several diagnostic characteristics that were statistically different, between the normal and the POTS groups. Initially, we looked at the following variables: gender, age, BMI, daily water intake, daily sleeping hours, history of motion sickness, family history of syncope and study BP, supine HR, supine systolic BP, supine diastolic BP, HR increment and maximum HR within the first 10 min after standing, and the systolic BP and the diastolic BP within the first three min after standing. We then tested whether there were any differences in the occurrence of above-mentioned indexes between the normal and the POTS groups. As expected, we found that the daily water intake and daily sleeping hours, supine HR, HR increment and maximum HR in the first 10 min after standing differed significantly between the two groups. After the Logistic regression analysis, three risk factors influencing the incidence of POTS were identified. The three risk factors included a faster supine HR, insufficient daily water intake and less sleeping hours.

Many studies indicate that it is important to drink sufficient water to avoid orthostatic intolerance [28]–[31]. Jarjour pointed out that water intake was the non-drug treatment choice for upright intolerance, and he suggested that 2–2.5 liter per day was needed [1]. 500 ml of water intake early in the morning before getting up can help by increasing the blood pressure within five min, and avoiding upright intolerance [29]. Mathias et al. showed that BP would not significantly change for normal young men after drinking of 500 ml of water, but for the elderly, 500 ml of water could increase their BP [30]. This might be related to the nervous system regulating the water baroreceptor, or be associated with the redistribution of water. Regarding the efficacy of water intake versus soup intake on the treatment of the intolerance symptoms in POTS, Z'Graggen et al. [32] found that no matter which drink was chosen, the intolerance symptoms improved. However, up to now, there has not been any analysis of the risk factors for developing POTS in the young. This present study revealed that the occurrence of POTS in children who had a water intake of less than 800 ml/day was 3.877 times higher than those whose water intake was more than 800 ml/day. There are several facts which may explain the role of water intake in preventing orthostatic intolerance. Firstly, water intake itself has a great pressor effect in autonomic failure. This effect could be antagonized by ganglionic blockade, and was associated with increases in plasma norepinephrine and in muscle sympathetic nerve activity [33]. Second, Shannon et al. observed that the HR increased less after water intake, but our understanding of this HR reduction is limited [29]. The fact that HR was reduced after water intake in the setting of upright raised the possibility of baroreflex modulation of HR. Such an effect could lead to lower cardiac sympathetic drive. Such a targeted decrease in sympathetic activity to the ventricles might improve upright tolerance, in keeping with the so called “ventricular theory” of the pathophysiology of syncope [28]. Thirdly, it was found that the faint was associated with a vasodilatation and a reduction in muscle sympathetic nerve activity together with an increase in plasma epinephrine. The fact of the total peripheral resistance increase in response to water intake might suggest that water somehow abrogates these vasodilator responses [28]. Lastly, the important factor contributing to inter-individual variation in response to upright posture is orthostatic loss of plasma volume. Water intake helps to increase the plasma volume and decreased the orthostatic intolerance.

A number of studies have found that children with POTS complained of being sleepy during the day, and of having a decreased quality of life [15], [16]. But, whether there would be any specific relationship between sleeping and POTS was unclear. This study revealed that people with eight hours of sleep or longer were less likely to develop symptoms of POTS. It was found in this study that with less than 8 hours of sleep/day, the risk of getting POTS was 5.905 times greater than those whose sleeping was longer than 8 hours/day. The reason of this result is not fully understood. Irwin et al. found that patients with insomnia had increased nocturnal catecholamine levels as compared with controls [34] which might contribute the pathophysiology of POTS. Additionally, insufficient sleep or sleep disruption is associated with significant increases in plasma cortisol levels [35]. In normal subjects, waking periods and stage-N1 sleep accompany cortisol increases, whereas slow-wave sleep is associated with declining plasma cortisol levels. Therefore, short of slow-wave sleep increases the lever of cortisol, which might induce to syndromes similar to POTS. Lastly, the possible explanation was that the sleep disturbance might relate with heightened sympathetic activation [36].

The present study indicated that if the supine HR was increased by 10 beats/min, the risk of POTS was 1.583 times. The causative physiological mechanisms were not clear. According to the B-J reflex [37], when baroreceptors detect a decrease in BP, efferent sympathetic activity increases. The increase in sympathetic tone enhances total peripheral vascular resistance, and it produces positive chronotropic and inotropic cardiac effects. When HR has inappropriately increased, the increased cardiac sympathetic stimulation, in a setting of ventricular hypovolemia, is thought to result in large pressure transients that are evoked by the contraction of the ventricular muscle on an “empty chamber”. The vigorous contraction of the hypovolemic ventricle, in turn, is thought to stimulate “ventricular afferents” in the left ventricle, resulting in vagal activation, inhibition of sympathetic activity, cardiac and circulatory collapse, and resultant syncope.

Understanding the risk factors for POTS would be helpful in both predicting and preventing the disease process. ROC analysis yielded a sensitivity of 80.5%, and a specificity of 75% in predicting POTS risk, by using the parameters mentioned above in the 600 children. And, when we applied the risk factors to the validation group, the results showed the sensitivity was 73.3% and the specificity was 72.5%. Thus, by timely detecting the risk factors, we were able to predict the possibility of the occurrence of POTS and give the parents of children and adolescents advice on avoiding the risk factors, thusly reducing the number of children with POTS.

The present study, however, has some limitations. The sample size, given the incidence of POTS, seemed still small. In the future a larger scaled investigation is needed. Although the area where the subjects came from was in the central China, the life habits and study situation of these children and adolescents in this area could not adequately represent all the children and adolescents in China. However, in the present study, we were able to provide a prevalence rate for POTS among children and adolescents and identify the risk factors for POTS in these children. As a result, clinicians, utilizing the paradigm presented here, can better predict, treat and prevent POTS.

## Supporting Information

Table S1 **Data of the test group.** HR1 to HR10 recorded the steady heart rate in each min during the up-right position in the head-up test; and 3 standing BPs at 3 minutes, 6 minutes and 9 minutes, respectively. The “highest HR” means the highest HR during the up-right position. “HR increase” means the highest HR minus supine HR. “Supine HR 10” means supine HR divided by 10. Car sick: “1” stands for “yes” and “0” stands for “no”. Family history: “1” stands for “yes” and “0” stands for “no”. Water intake: “1” stands for less than 800 ml/day and “0” stands for more than 800 ml/day. Sleeping hours: “1” stands for less than 8 h/day and “0” stands for longer than 8 h/day. School-induced burden: “1” stands for “yes” and “0” stands for “no”. POTS: “1” stands for patient and “0” stands for non-patient. Sex: “1” stands for male and “2” stands for female. BMI: body mass index = weight(kg)/height(m)^2^.
(PDF)

Table S2 **Data of the validation group.** HR1 to HR10 recorded the steady heart rate in each min during the up-right position in the head-up test and 3 standing BPs at 3 minutes, 6 minutes and 9 minutes, respectively. The “highest HR” means the highest HR during the up-right position. “HR increase” means the highest HR minus supine HR. “supine HR 10” means “supine HR” divided by 10. Car sick: “1” stands for “yes” and “0” stands for “no”. Family history: “1” stands for “yes” and “0” stands for “no”. Water intake: “1” stands for less than 800 ml/day and 0 stands for more than 800 ml/day. Sleeping hours: “1” stands for less than 8 h/day and “0” stands for more than 8 h/day. School-induced burden: “1” stands for “yes” and 0 stands for “no”. POTS: “1” stands for patient and “0” stands for non-patient. Sex: “1” stands for male and “2” stands for female. BMI: body mass index = weight(kg)/height(m)^2^.
(PDF)
