# Supplementary material for: Risk Factors for Postural Tachycardia Syndrome in Children and Adolescents
Source: PLoS One. 2014 Dec 4;9(12):e113625. doi: 10.1371/journal.pone.0113625 (PMC4256207; doi:10.1371/journal.pone.0113625)
Supplement: Table S1 — Data of the test group. HR1 to HR10 recorded the steady heart rate in each min during the up-right position in the head-up test; and 3 standing BPs at 3 minutes, 6 minutes and 9 minutes, respectively. The “highest HR” means the highest HR during the up-right position. “HR increase” means the highest HR minus supine HR. “Supine HR 10” means supine HR divided by 10. Car sick: “1” stands for “yes” and “0” stands for “no”. Family history: “1” stands for “yes” and “0” stands for “no”. Water intake: “1” stands for less than 800 ml/day and “0” stands for more than 800 ml/day. Sleeping hours: “1” stands for less than 8 h/day and “0” stands for longer than 8 h/day. School-induced burden: “1” stands for “yes” and “0” stands for “no”. POTS: “1” stands for patient and “0” stands for non-patient. Sex: “1” stands for male and “2” stands for female. BMI: body mass index = weight(kg)/height(m)2. (PDF) [file pone.0113625.s001.pdf]

Test group

code	sex	age	height	weight	BMI	supine HR	supine SBP	supine DBP	HR1	HR2	HR3	SBP3	DBP3	HR4	HR5	HR6	SBP6	DBP6	HR7	HR8	HR9	SBP9	DBP9	HR10	highest HR	car sick	family history	water intake	sleeping hours	school-indiced burden	HR increase	supine HR10	POTS
4	2	16	166	55	19.96	74	115	79	95	95	100	117	87	95	100	101	116	82	102	102	98	116	81	100	102	1	0	0	0	0	28	7.40	0
5	2	16	162	50	19.05	72	111	73	91	87	93	113	78	94	90	93	112	76	96	90	86	115	78	83	96	1	0	0	0	1	24	7.20	0
6	2	16	158	52	20.83	73	94	59	88	81	96	107	74	80	89	90	94	64	85	87	87	102	65	93	96	1	0	1	0	0	23	7.30	0
9	2	17	162	62	23.62	72	117	78	71	78	80	120	78	75	77	72	148	80	74	83	79	115	77	82	83	1	0	0	1	1	11	7.20	0
18	2	16	155	60	24.97	90	114	74	85	87	86	110	73	86	86	88	107	71	87	92	88	107	70	88	92	1	0	0	1	1	2	9.00	0
21	2	17	162	57	21.72	85	118	70	117	110	105	135	79	105	116	110	117	84	105	105	105	131	78	111	117	0	0	1	0	0	32	8.50	0
23	2	12	164	60	22.31	70	115	72	76	74	80	116	76	74	72	80	113	78	77	74	75	114	76	80	80	0	1	1	1	1	10	7.00	0
26	1	12	155	45	18.73	91	127	78	112	108	105	144	99	105	104	102	141	103	105	104	109	139	101	109	112	1	1	0	0	1	21	9.10	0
27	2	17	159	56	22.15	85	121	61	78	94	93	106	64	96	93	95	113	63	96	95	97	113	64	96	97	1	0	0	0	0	12	8.50	0
30	2	18	162	47	17.91	68	118	71	91	91	90	122	80	85	84	91	118	75	87	95	86	110	74	95	95	0	0	0	0	0	27	6.80	0
31	2	16	159	47	18.59	72	103	61	95	93	88	111	73	85	94	87	104	69	93	92	96	110	62	94	96	1	0	1	0	1	24	7.20	0
32A	2	16	165	53	19.47	82	119	72	89	92	86	116	69	85	91	91	106	68	87	92	86	111	69	89	92	0	0	0	1	1	10	8.20	0
36A	2	12	154	44	18.55	96	99	66	115	116	115	115	83	113	121	118	110	80	123	121	123	108	80	124	124	1	0	1	0	1	28	9.60	1
37	2	16	160	58	22.66	76	121	73	86	85	90	124	89	97	100	93	121	80	93	92	98	123	80	93	100	1	0	0	0	1	24	7.60	0
38	2	17	155	45	18.73	85	127	74	93	98	100	139	96	100	100	104	123	85	98	103	103	135	91	94	104	0	0	0	1	1	19	8.50	0
39	1	12	161	64	24.69	94	126	84	105	112	111	131	95	107	110	109	134	97	111	112	118	131	95	110	118	0	0	1	0	0	24	9.40	0
40A	2	16	160	48	18.75	106	110	69	106	113	112	122	79	109	113	111	113	76	109	110	108	118	76	106	113	0	1	0	0	0	7	10.60	0
41A	2	16	153	50	21.36	68	131	85	75	74	76	140	82	74	75	75	130	75	77	75	71	137	47	75	77	1	0	1	0	1	9	6.80	0
42	2	14	160	44	17.19	62	111	58	68	55	54	115	71	55	59	56	108	68	58	59	63	112	71	61	68	1	0	1	0	1	6	6.20	0
43	2	12	160	42	16.41	98	112	68	119	118	124	125	74	125	122	128	122	80	129	129	125	137	83	124	129	1	0	0	1	1	31	9.80	1
48A	2	17	155	58	24.14	74	113	63	101	100	104	116	74	101	101	107	112	74	102	115	116	115	78	111	116	1	0	1	1	0	42	7.40	1
49	2	16	163	47	17.69	79	104	57	99	97	91	117	68	95	88	98	105	73	98	97	98	102	67	94	99	1	1	1	0	0	20	7.90	0
50	2	16	166	62	22.50	89	103	70	96	105	100	107	74	106	103	106	108	70	104	98	103	109	64	105	106	1	0	0	1	0	17	8.90	0
51A	2	16	164	90	33.46	86	111	73	105	112	104	104	76	109	107	110	109	76	109	113	111	113	76	108	113	1	0	0	1	1	27	8.60	0
53	1	12	135	30	16.46	87	115	77	113	103	103	127	75	105	104	107	130	85	104	101	97	112	70	104	113	1	0	0	0	1	26	8.70	0
54A	2	12	152	39	16.88	92	122	78	99	100	99	125	91	95	97	102	124	89	98	104	92	128	85	98	104	0	1	0	0	0	12	9.20	0
55	2	17	163	49	18.44	65	106	66	84	89	93	109	79	91	93	96	103	75	96	104	100	117	76	96	104	1	0	1	0	1	39	6.50	0
59	2	16	164	44	16.36	89	102	60	124	108	118	107	69	121	117	115	100	65	124	121	122	99	64	122	124	1	0	1	1	1	35	8.90	1
60A	2	15	165	59	21.67	61	114	72	69	74	69	129	84	72	77	68	121	78	78	73	75	121	83	75	78	1	0	0	1	1	17	6.10	0
62	2	17	152	45	19.48	84	105	60	89	84	93	98	62	85	92	93	101	62	93	95	99	104	56	90	99	0	0	1	0	0	15	8.40	0
63	2	16	157	60	24.34	89	118	81	85	94	93	120	86	97	90	92	109	76	95	95	94	107	75	95	95	1	0	0	1	1	6	8.90	0
64	2	16	164	49	18.22	58	118	67	73	82	74	114	70	75	77	73	116	73	77	76	79	116	73	81	82	0	0	1	1	1	24	5.80	0
65	2	16	158	48	19.23	66	105	62	82	83	80	112	72	81	84	84	108	73	81	82	86	115	78	82	86	1	1	1	0	0	20	6.60	0
69	2	16	161	55	21.22	75	103	64	87	79	89	116	76	82	81	83	114	77	84	86	82	109	76	83	89	1	0	1	0	1	14	7.50	0
72	2	18	168	53	18.78	62	101	63	77	83	87	103	66	88	95	93	98	63	93	97	92	106	62	91	97	0	0	1	1	1	35	6.20	0
76	2	18	160	41	16.02	72	94	61	105	106	113	104	68	109	119	113	97	65	114	113	106	101	71	107	119	1	0	1	1	1	47	7.20	1
77	2	16	150	50	22.22	67	117	72	68	72	72	122	73	77	77	84	119	76	73	74	79	129	86	78	84	0	0	0	0	0	17	6.70	0
78	2	15	155	44	18.31	77	112	75	75	85	88	124	86	90	96	93	120	81	88	93	95	116	83	87	96	1	0	1	0	1	19	7.70	0
79	2	18	162	47	17.91	78	110	65	84	85	83	127	78	85	83	87	115	70	87	93	88	111	77	86	93	1	1	1	0	0	15	7.80	0
80	2	16	159	56	22.15	64	101	57	83	78	75	116	79	76	91	82	105	71	86	92	90	117	75	88	92	0	0	0	0	1	28	6.40	0
81	2	18	164	75	27.89	80	111	69	88	93	94	114	76	89	88	88	108	67	85	87	87	111	64	87	94	1	0	0	0	0	14	8.00	0
82	2	17	157	68	27.59	81	134	77	81	81	87	133	79	87	89	84	134	88	86	88	83	124	84	88	89	1	0	0	0	1	8	8.10	0
83A	2	14	150	48	21.33	86	113	66	100	96	95	123	78	96	97	94	114	77	103	102	93	115	73	91	103	0	1	0	0	0	17	8.60	0
85A	2	16	162	54	20.58	75	113	64	87	84	81	111	67	86	94	96	109	65	86	84	87	103	62	91	96	0	0	0	0	0	21	7.50	0
86	2	16	161	63	24.30	82	114	69	92	96	99	118	76	98	97	97	115	82	95	94	98	119	78	98	99	0	0	1	0	0	17	8.20	0
87A	2	18	163	57	21.45	63	113	70	90	87	91	119	78	91	94	90	114	76	88	84	84	110	75	84	94	1	0	0	0	0	31	6.30	0
88A	2	18	163	53	19.95	71	117	70	87	87	83	130	86	80	84	84	122	82	86	83	88	125	80	84	87	0	0	0	0	0	16	7.10	0
89	2	16	162	54	20.58	50	121	73	62	58	65	126	79	73	72	64	123	78	72	76	65	124	81	70	76	1	0	0	0	0	26	5.00	0
90	2	16	163	57	21.45	72	106	69	90	85	88	110	77	89	91	90	111	71	97	96	92	105	73	91	97	0	0	1	0	0	25	7.20	0
91A	2	16	160	45	17.58	107	114	76	107	119	117	125	89	119	115	125	129	84	121	116	115	128	88	114	125	0	0	1	0	0	18	10.70	0
92	2	15	159	48	18.99	72	113	73	93	77	82	119	77	88	84	82	117	76	91	96	92	118	75	90	96	1	1	0	0	1	24	7.20	0
93	2	17	164	50	18.59	85	110	70	91	90	93	119	77	92	98	99	113	75	90	94	95	118	74	97	99	1	1	1	0	1	14	8.50	0
94A	2	15	157	43	17.44	80	100	69	90	90	89	119	83	91	96	95	139	96	80	90	94	121	77	97	97	1	1	0	0	0	17	8.00	0
95	2	15	156	47	19.31	82	120	81	73	85	90	142	96	90	84	90	126	92	75	84	79	125	85	82	90	1	1	1	0	0	8	8.20	0
96	2	17	159	54	21.36	67	106	66	60	75	77	118	85	76	77	76	113	81	75	73	73	109	79	79	79	0	0	1	0	0	12	6.70	0

Test group

code	sex	age	height	weight	BMI	supine HR	supine SBP	supine DBP	HR1	HR2	HR3	SBP3	DBP3	HR4	HR5	HR6	SBP6	DBP6	HR7	HR8	HR9	SBP9	DBP9	HR10	highest HR	car sick	family history	water intake	sleeping hours	school-indiced burden	HR increase	supine HR10	POTS
97A	2	16	168	66	23.38	79	112	62	89	96	94	118	67	95	93	89	118	66	87	92	90	110	64	94	94	0	0	0	0	0	15	7.90	0
98	2	18	162	56	21.34	92	118	78	110	104	103	131	78	107	108	104	117	79	98	97	91	118	80	100	110	0	0	0	0	0	18	9.20	0
101A	2	17	157	45	18.26	92	109	72	98	101	94	113	75	98	106	97	116	78	100	102	99	115	72	98	106	0	0	1	0	1	14	9.20	0
104	2	18	157	44	17.85	82	103	62	103	119	108	107	67	104	96	98	100	66	100	102	104	103	66	92	119	0	0	1	0	0	37	8.20	0
108A	2	16	162	51	19.43	78	101	65	85	87	68	117	78	71	77	74	122	78	81	82	79	107	72	70	87	1	0	1	0	0	9	7.80	0
110A	2	17	159	48	18.99	90	116	66	92	98	108	121	64	102	102	108	121	60	95	98	102	119	55	100	108	1	0	1	0	0	18	9.00	0
111	2	11	160	94	36.72	84	118	66	111	102	113	134	89	104	112	95	133	84	107	101	103	124	82	104	113	1	0	1	0	1	29	8.40	0
112	2	12	153	44	18.80	87	115	76	98	99	95	126	79	99	101	103	114	73	106	105	109	120	75	106	109	0	1	0	0	0	22	8.70	0
114A	2	16	160	53	20.70	85	128	71	96	102	103	129	80	100	110	110	122	75	106	110	105	124	73	106	110	1	0	1	1	0	25	8.50	0
116	2	17	159	60	23.73	65	121	71	105	111	100	123	72	109	110	112	128	83	114	110	101	127	82	96	114	0	0	1	1	0	49	6.50	1
117A	2	14	149	43	19.37	63	108	65	79	73	80	115	72	82	85	94	108	68	91	94	94	109	63	90	94	0	0	0	0	0	31	6.30	0
119	2	17	157	56	22.72	79	104	69	79	72	74	104	71	76	85	75	100	66	83	87	79	99	64	77	87	0	0	0	0	0	8	7.90	0
121	2	15	154	44	18.55	89	115	72	98	110	81	129	96	90	92	96	124	76	94	101	95	123	84	94	110	1	0	0	0	0	21	8.90	0
124A	2	18	163	54	20.32	80	120	74	86	87	91	131	76	83	84	95	132	85	90	92	90	129	81	92	95	1	0	1	0	0	15	8.00	0
125	2	16	169	53	18.56	85	108	63	119	116	120	119	81	120	124	121	124	77	124	107	116	123	77	108	124	1	0	1	0	0	39	8.50	0
127	2	12	149	42	18.92	75	115	52	94	101	96	121	65	107	105	110	108	67	112	107	105	106	67	107	112	1	0	1	0	0	37	7.50	0
128A	2	17	154	41	17.29	74	108	69	100	103	97	111	76	102	110	103	111	77	103	103	94	122	85	94	110	1	0	1	1	1	36	7.40	0
129A	2	16	162	54	20.58	91	112	71	108	103	113	110	81	108	114	110	111	83	118	116	118	126	89	114	118	1	0	1	1	0	27	9.10	0
131	2	16	163	58	21.83	72	115	68	78	82	89	120	76	88	93	100	122	85	90	91	95	115	75	103	103	1	0	1	0	1	31	7.20	0
132	2	17	162	49	18.67	87	116	75	76	81	85	120	74	93	88	99	117	81	95	90	84	125	82	86	99	0	0	1	1	0	12	8.70	0
133	2	16	163	59	22.21	72	109	69	84	78	74	115	81	77	85	83	109	76	86	86	84	112	84	80	86	1	0	1	1	0	14	7.20	0
134	2	16	151	41	17.98	63	108	75	73	73	87	113	82	81	91	81	116	87	78	90	82	112	84	88	91	1	0	0	0	0	28	6.30	0
135	2	12	153	44	18.80	84	116	72	110	105	110	116	62	105	108	109	116	69	115	115	108	106	60	113	115	0	0	0	0	1	31	8.40	0
139A	2	17	162	54	20.58	65	103	60	89	79	88	109	68	78	86	80	114	74	81	84	76	108	69	78	89	1	1	1	1	1	24	6.50	0
141	1	13	162	50	19.05	86	125	73	98	100	102	136	78	90	95	100	129	85	97	100	103	136	79		103	0	0	0	0	0	17	8.60	0
143	2	15	163	61	22.96	81	117	77	80	76	76	120	80	80	92	79	116	77	91	85	80	118	73	81	92	1	0	1	0	1	11	8.10	0
144	2	15	162	48	18.29	65	110	64	64	72	84	115	67	91	102	89	120	79	89	70	72	110	75	92	102	0	1	1	0	0	37	6.50	0
145	1	13	162	53	20.20	98	121	76	120	116	122	148	85	125	125	123	131	87	130	136	138	127	84	134	138	0	1	0	0	0	40	9.80	1
146	2	12	155	44	18.31	85	107	70	85	88	88	120	88	90	89	93	122	88	96	96	94	121	83	96	96	0	0	0	0	1	11	8.50	0
148	1	12	158	70	28.04	100	124	65	117	107	108	125	59	108	110	102	121	65	111	111	111	128	63	111	117	1	0	1	0	0	17	10.00	0
149	1	12	152	44	19.04	85	117	82	93	84	88	123	86	90	96	109	118	86	105	108	109	131	82	94	109	0	0	0	0	1	24	8.50	0
151	2	16	165	59	21.67	66	112	64	75	86	81	109	78	87	91	82	114	75	84	85	88	114	80	85	91	0	0	0	0	0	25	6.60	0
153	2	17	155	49	20.40	69	103	68	70	71	71	115	77	70	75	78	113	76	70	75	71	112	75	77	78	1	0	0	0	0	9	6.90	0
154	2	16	158	49	19.63	70	100	65	84	79	84	113	82	86	84	84	109	77	79	82	85	106	70	83	86	1	0	0	0	0	16	7.00	0
164	2	18	161	53	20.45	82	104	64	93	109	97	111	76	95	98	90	119	73	96	104	99	111	75	95	109	1	0	1	0	0	27	8.20	0
165A	2	17	155	55	22.89	75	104	64	75	88	91	112	72	87	85	82	104	67	88	87	84	112	71	78	91	1	0	1	0	1	16	7.50	0
166	2	17	158	90	36.05	73	106	69	68	85	80	116	84	88	86	90	117	83	87	91	89	116	79	97	97	1	0	1	0	0	24	7.30	0
168	2	16	166	51	18.51	72	96	63	75	81	83	104	72	89	86	89	107	70	88	89	90	106	73	88	90	0	1	1	0	0	18	7.20	0
169	2	17	160	54	21.09	73	108	63	88	83	81	113	84	84	84	83	111	79	87	86	85	120	82	84	88	1	0	1	0	0	15	7.30	0
170A	2	16	161	56	21.60	82	114	71	120	100	97	122	77	101	96	92	116	75	95	91	99	120	72	92	120	1	0	1	0	1	38	8.20	0
171	2	16	159	52	20.57	88	126	80	98	89	82	126	84	90	89	85	129	80	84	90	87	126	83	104	104	0	0	1	1	0	16	8.80	0
172	2	17	163	52	19.57	77	97	67	96	99	96	111	83	96	99	98	115	85	98	98	97	107	80	96	99	0	1	1	0	1	22	7.70	0
173A	1	13	165	45	16.53	86	117	71	113	106	102	117	84	111	107	114	118	82	102	106	107	113	79	110	114	0	0	0	0	1	28	8.60	0
175	2	18	153	41	17.51	82	107	71	90	92	94	115	78	93	96	94	115	80	86	97	89	115	80	93	97	0	0	0	0	0	15	8.20	0
176A	1	14	156	45	18.49	97	108	71	105	112	113	119	84	108	108	111	119	83	112	106	114	117	85	112	114	0	1	1	0	1	17	9.70	0
178	2	16	153	61	26.06	82	98	59	93	83	89	112	75	88	91	90	114	73	97	93	89	112	71	87	97	1	0	1	0	0	15	8.20	0
184A	1	13	149	39	17.57	72	101	66	76	75	84	110	78	80	90	85	109	77	86	93	87	120	79	84	93	1	0	0	0	1	21	7.20	0
185	1	17	178	65	20.52	75	117	67	87	90	89	130	77	92	93	97	127	72	99	98	96	135	72	96	99	0	0	0	0	0	24	7.50	0
186	1	13	165	50	18.37	64	105	64	80	89	81	118	82	83	85	80	121	88	90	84	87	112	79	94	94	1	0	0	0	1	30	6.40	0
188	2	13	146	45	21.11	89	115	70	103	113	104	128	80	109	105	101	122	77	111	106	109	123	79	108	113	1	1	1	0	1	24	8.90	0
190A	1	18	169	61	21.36	64	128	77	70	71	74	126	85	70	72	75	135	84	75	76	74	130	89	75	76	0	0	1	1	0	12	6.40	0
191	1	15	162	48	18.29	70	103	63	83	85	79	118	75	84	86	84	110	75	83	85	83	104	67	84	86	0	0	0	0	0	16	7.00	0
196	1	14	169	54	18.91	80	110	70	95	100	86	117	73	98	95	98	114	74	99	102	102	117	77	98	102	1	1	0	0	1	22	8.00	0
197	2	12	160	48	18.75	88	117	76	97	96	96	125	85	93	97	103	124	84	94	95	98	122	82	100	103	0	0	0	0	1	15	8.80	0
198	1	14	168	52	18.42	80	113	67	95	88	94	126	82	96	97	97	117	79	101	103	103	123	78	100	103	1	1	1	0	1	23	8.00	0

Test group

code	sex	age	height	weight	BMI	supine HR	supine SBP	supine DBP	HR1	HR2	HR3	SBP3	DBP3	HR4	HR5	HR6	SBP6	DBP6	HR7	HR8	HR9	SBP9	DBP9	HR10	highest HR	car sick	family history	water intake	sleeping hours	school-indiced burden	HR increase	supine HR10	POTS
199	1	14	161	44	16.97	86	110	49	105	99	103	116	73	102	98	104	117	60	110	111	110	116	66	118	118	1	1	1	0	1	32	8.60	0
201	2	13	164	55	20.45	70	107	59	80	88	81	118	76	75	81	86	115	69	88	78	88	114	66	84	88	1	1	0	0	1	18	7.00	0
202	2	14	151	51	22.37	97	117	71	105	107	115	129	93	118	123	120	126	93	122	112	120	134	89	119	123	1	1	1	0	1	26	9.70	1
204	2	13	144	34	16.40	95	101	56	115	114	114	105	62	110	120	107	106	67	108	109	113	107	62	114	120	0	0	0	0	1	25	9.50	1
205	2	13	163	54	20.32	98	101	59	111	111	112	120	78	109	112	110	114	74	108	118	115	111	78	108	118	1	1	0	0	1	20	9.80	0
206	2	13	163	50	18.82	86	124	78	88	93	90	138	88	112	94	91	132	87	95	102	102	131	82	106	112	0	1	1	0	1	26	8.60	0
207	1	15	171	62	21.20	84	111	55	106	111	98	120	77	101	108	108	119	75	101	107	108	119	75	104	111	1	0	0	0	0	27	8.40	0
208	2	14	153	58	24.78	94	113	74	87	97	101	119	77	96	102	100	118	77	103	97	118	118	80	96	118	0	1	0	0	1	24	9.40	0
209	1	13	173	55	18.38	93	116	73	112	103	111	124	86	100	100	99	119	85	117	114	113	121	79	109	117	1	0	1	0	1	24	9.30	0
211	2	13	162	45	17.15	95	104	64		102	118	135	79	112	117	111	137	85	103	110	113	132	81	116	118	0	1	1	1	1	23	9.50	0
212	1	15	175	66	21.55	80	119	65	113	104	103	125	81	108	103	113	127	81	108	116	100	127	83	106	116	0	0	0	0	0	36	8.00	0
215	1	13	155	46	19.15	81	116	62	89	95	97	121	74	99	96	101	115	77	93	97	96	115	68	93	101	0	0	0	0	0	20	8.10	0
217	1	13	176	51	16.46	72	125	71	100	103	109	139	94	100	103	108	129	86	104	104	106	129	83	113	113	1	0	1	0	1	41	7.20	1
218	1	13	160	48	18.75	87	115	64	113	118	109	127	79	108	105	112	130	83	120	109	121	128	76	110	121	0	0	0	0	1	34	8.70	1
220	2	13	155	53	22.06	99	127	84	118	110	108	141	98	109	108	114	140	74	108	112	110	139	85	109	118	0	0	1	0	0	19	9.90	0
223	2	13	159	53	20.96	77	111	60	90	88	94	117	67	90	92	97	117	71	94	96	94	131	74	94	97	1	1	1	0	1	20	7.70	0
224	1	13	156	43	17.67	112	110	66	122	121	113	114	79	115	117	111	116	75	111	116	112	114	65	110	122	0	0	0	0	1	10	11.20	0
225	2	13	170	58	20.07	87	121	76	107	109	104	131	98	108	110	107	129	93	111	111	107	126	82	108	111	0	0	0	0	1	24	8.70	0
228	2	14	154	47	19.82	85	118	81	90	92	97	134	95	102	98	103	132	79	105	109	106	132	92	100	109	1	1	1	0	1	24	8.50	0
229	2	14	168	55	19.49	84	118	76	85	94	96	136	87	100	94	99	125	78	94	96	87	121	83	93	100	0	0	0	0	1	16	8.40	0
231	2	15	157	55	22.31	84	125	84	80	89	90	137	96	90	94	93	141	91	97	93	95	146	98	99	99	0	0	0	0	1	15	8.40	0
232	1	14	176	68	21.95	92	123	75	104	102	100	131	80	98	97	107	131	80	102	105	95	129	78	97	105	1	0	0	1	1	13	9.20	0
236	1	14	167	62	22.23	93	113	74	112	123	102	122	89	100	104	104	122	83	103	116	102	129	75	99	123	1	0	0	0	1	30	9.30	1
237	1	13	172	55	18.59	90	134	85	108	110	112	154	105	118	114	120	156	106	116	116	114	143	98	120	120	1	1	1	0	1	30	9.00	0
240	2	13	155	45	18.73	88	105	76	106	107	108	116	77	114	116	107	112	77	107	112	114	115	76	122	122	0	0	0	0	0	34	8.80	0
241	2	14	158	50	20.03	85	129	74	111	81	85	125	58	86	91	90	124	68	87	88	89	125	55	89	111	0	1	1	0	1	26	8.50	0
242	1	13	168	59	20.90	75	112	58	112	105	107	133	76	106	103	107	125	70	116	116	112	118	70	111	116	1	1	1	1	0	41	7.50	1
243	1	13	158	42	16.82	85	116	61	102	107	105	109	72	102	97	105	112	70	112	114	100	113	74	103	114	1	1	0	0	1	29	8.50	0
244	2	12	154	50	21.08	86	111	76	101	97	100	119	81	111	100	99	122	79	100	104	108	114	77	98	111	1	0	1	0	1	25	8.60	0
247	1	13	158	43	17.22	88	109	69	107	110	111	126	84	93	120	118	112	73	125	121	121	125	71	121	125	1	0	0	0	1	37	8.80	0
248	1	13	174	52	17.18	90	124	79	104	109	103	127	75	108	110	111	121	74	116	118	110	130	77	114	118	1	0	1	0	0	28	9.00	0
249	1	13	165	53	19.47	79	123	57	94	101	88	119	64	92	95	100	127	67	101	88	87	129	65	92	101	1	1	1	0	1	22	7.90	0
250	2	13	150	50	22.22	70	99	62	87	74	76	106	70	82	83	82	101	72	87	85	81	106	71	89	89	0	0	1	0	1	19	7.00	0
251	1	18	174	65	21.47	77	117	70	85	82	85	118	77	85	90	89	119	76	89	85	88	116	76	86	90	0	0	1	1	0	13	7.70	0
252	2	13	160	40	15.63	122	126	81	133	127	133	139	98	133	131	133	133	94	133	133	135	147	98	134	135	1	0	1	0	1	13	12.20	0
254	2	14	158	45	18.03	92	117	68	104	103	100	121	77	102	104	102	117	83	103	102	108	111	79	104	108	0	1	1	0	0	16	9.20	0
255	1	18	166	55	19.96	80	119	64	77	81	79	122	87	84	84	83	126	83	88	79	79	126	78	78	88	0	0	1	0	0	8	8.00	0
257	1	15	172	57	19.27	76	121	81	87	88	92	124	81	99	94	95	119	87	97	90	93	122	84	96	99	1	0	0	0	1	23	7.60	0
259	1	14	168	64	22.68	76	116	43	96	90	100	119	64	95	103	105	126	76	99	97	105	124	67	96	105	1	1	0	0	1	29	7.60	0
260	1	14	168	53	18.78	80	104	67	111	101	98	101	71	101	102	104	108	70	97	102	94	106	72	104	111	1	1	0	0	1	31	8.00	0
262	1	15	172	70	23.66	84	105	62	98	102	99	117	79	96	98	97	118	77	96	98	92	120	75	97	102	1	0	0	0	1	18	8.40	0
263	1	13	175	55	17.96	75	110	68	85	90	94	127	82	92	88	96	121	80	86	96	98	119	78	102	102	0	0	0	0	1	27	7.50	0
264	2	15	162	50	19.05	75	102	66	81	82	85	111	78	89	88	84	108	73	85	87	85	107	68	87	89	1	1	1	0	1	14	7.50	0
265	2	15	170	55	19.03	65	109	66	86	80	84	111	75	92	87	89	109	70	88	92	89	107	71	88	92	1	1	0	0	1	27	6.50	0
266	2	13	160	45	17.58	92	110	67	102	113	106	114	76	113	116	109	125	75	119	125	119	116	71	121	125	1	1	1	1	1	33	9.20	1
267	1	13	170	55	19.03	86	119	74	92	93	102	126	92	104	94	100	131	81	98	93	91	142	90	90	104	1	0	0	0	1	18	8.60	0
268	2	13	157	40	16.23	105	110	62	115	114	120	114	75	114	115	118	114	73	116	120	120	119	72	113	120	1	1	1	0	1	15	10.50	1
269	1	13	167	51	18.43	74	99	54	94	99	91	112	70	95	96	98	109	67	98	97	95	111	74	97	99	0	0	0	0	0	25	7.40	0
271	2	13	160	46	17.97	81	99	67	89	91	91	115	91	92	93	93	123	84	92	86	93	128	88	92	93	1	1	1	1	1	12	8.10	0
272	1	14	163	46	17.31	65	118	67	101	103	107	118	73	99	105	97	117	71	112	117	106	117	75	114	117	0	1	1	1	0	52	6.50	1
273	1	13	166	65	23.59	64	128	69	84	88	91	137	85	85	87	90	144	82	88	90	86	125	88	83	91	1	1	0	0	1	27	6.40	0
275	1	14	150	62	27.56	84	127	66	96	95	96	134	78	103	96	97	130	87	102	99	97	127	77	99	103	1	1	0	0	1	19	8.40	0
277	1	13	158	50	20.03	90	100	56	96	102	99	108	60	96	98	94	103	62	100	100	108	99	62	102	108	0	1	1	0	1	18	9.00	0
278	2	14	168	78	27.64	78	126	75	83	85	87	129	85	85	91	87	127	94	88	94	87	124	84	79	94	1	0	1	0	1	16	7.80	0
280	2	14	156	45	18.49	75	95	63	83	78	82	103	77	84	85	88	108	75	74	81	82	103	75	84	88	1	0	1	0	1	13	7.50	0

Test group

code	sex	age	height	weight	BMI	supine HR	supine SBP	supine DBP	HR1	HR2	HR3	SBP3	DBP3	HR4	HR5	HR6	SBP6	DBP6	HR7	HR8	HR9	SBP9	DBP9	HR10	highest HR	car sick	family history	water intake	sleeping hours	school-indiced burden	HR increase	supine HR10	POTS
281	1	13	169	50	17.51	96	120	78	116	107	109	128	80	114	117	118	128	82	117	117	123	130	85	128	128	1	0	0	0	1	32	9.60	0
285	2	13	156	43	17.67	87	105	66	100	97	98	116	82	95	97	94	114	79	94	95	102	112	79	96	102	0	0	1	1	1	15	8.70	0
286	2	13	156	43	17.67	93	107	67	109	107	110	111	77	118	107	106	113	72	112	109	105	110	74	111	118	1	0	1	0	1	25	9.30	0
287	2	13	168	65	23.03	83	121	61	106	104	101	128	81	106	102	108	115	81	110	103	103	126	81	105	110	0	1	0	0	1	27	8.30	0
288	2	13	161	40	15.43	83	107	70	106	107	105	115	83	111	100	112	124	79	107	109	101	118	79	101	111	1	1	1	0	1	28	8.30	0
289	2	14	153	46	19.65	92	108	76	105	108	105	116	86	103	107	106	120	86	112	112	110	123	81	108	112	1	1	0	0	1	20	9.20	0
290	1	13	170	52	17.99	65	103	64	86	87	92	110	73	90	94	89	110	75	96	98	97	110	74	95	98	1	1	1	0	1	33	6.50	0
291	2	13	154	43	18.13	104	125	74	114	116	122	130	78	119	122	111	131	71	120	121	125	135	60	128	128	1	1	1	0	0	24	10.40	1
293	1	14	153	45	19.22	95	105	66	103	105	106	119	71	107	108	108	122	58	100	106	115	109	63	112	115	0	0	0	0	1	20	9.50	0
294	1	14	166	60	21.77	72	109	58	85	80	76	118	71	80	81	81	113	70	78	81	77	111	67	72	85	1	0	0	0	1	13	7.20	0
295	1	12	158	54	21.63	94	129	80	101	100	95	132	82	99	103	103	133	76	99	99	101	134	77	104	104	1	1	0	0	0	10	9.40	0
297	1	15	172	62	20.96	72	94	50	92	96	94	108	68	88	91	89	114	72	90	89	91	112	70	93	96	0	0	0	0	1	24	7.20	0
299	2	13	148	40	18.26	87	114	70	85	95	87	116	79	90	86	84	117	76	86	96	84	116	78	87	96	0	0	1	0	1	9	8.70	0
301	1	14	170	55	19.03	77	119	62	98	89	90	114	69	92	94	94	113	69	97	96	100	109	70	96	100	0	0	1	1	1	23	7.70	0
302	2	13	162	53	20.20	86	122	80	112	93	95	126	88	96	105	97	124	77	102	100	97	123	78	101	112	0	0	0	0	1	26	8.60	0
303	2	13	154	45	18.97	101	98	62	112	112	113	108	67	108	111	110	109	67	113	110	109	115	66	106	113	1	0	1	0	1	12	10.10	0
304	2	13	170	75	25.95	86	118	66	93	94	93	134	58	99	98	102	116	70	100	102	111	122	70	103	111	0	1	0	0	0	25	8.60	0
305	2	14	166	45	16.33	84	115	74	93	86	88	121	84	90	90	85	119	72	93	89	101	117	80	102	102	0	0	0	0	1	18	8.40	0
307	2	13	156	46	18.90	85	123	74	105	100	98	132	74	99	94	96	136	71	101	106	100	130	77	98	106	1	0	0	0	1	21	8.50	0
308	2	14	157	62	25.15	82	118	81	107	112	114	131	81	118	116	118	125	85	120	121	118	128	82	127	127	1	1	0	1	1	45	8.20	1
309	1	14	165	56	20.57	84	113	70	101	104	103	125	86	104	104	102	123	82	105	103	105	112	77	103	105	1	0	1	0	1	21	8.40	0
312	1	13	160	66	25.78	76	114	66	91	87	91	122	74	80	87	86	122	74	87	87	89	119	70	90	91	1	0	1	0	1	15	7.60	0
314	1	13	153	45	19.22	76	131	62	104	96	103	151	68	101	117	112	145	57	102	117	107	142	74	108	117	1	0	1	0	1	41	7.60	1
315	1	13	166	65	23.59	81	123	61	102	101	94	131	83	101	103	103	134	84	108	103	101	138	85	100	108	1	0	0	0	1	27	8.10	0
400	1	12	150	38	16.89	82	137	89	89	86	81			90	85	89			85	88	86			90	90	0	0	0	0	1	8	8.20	0
401	1	13	135	30	16.46	82	114	71	80	80	84	119	83	88	90	82	121	82	82	85	97	119	89	88	97	1	0	0	0	1	15	8.20	0
402	1	11	155	41	17.07	78	99	62	80	87	97	113	72	90	93	89	112	67	87	87	95	110	65	91	97	0	0	0	0	0	19	7.80	0
403	1	12	155	40	16.65	88	96	54	94	100	98	113	74	104	101	103	114	65	103	101	104	113	65	104	104	1	0	0	0	1	16	8.80	0
404	1	11	152	52	22.51	92	110	65	100	102	99	125	65	101	97	94	132	82	97	100	96	131	72	98	102	0	0	0	1	0	10	9.20	0
405	1	11	168	63	22.32	98	109	48	104	108	102	125	79	106	109	106	124	64	111	116	112	119	76	107	116	1	0	0	0	1	18	9.80	0
406	1	11	146	40	18.77	79	102	63	99	97	107	115	75	100	107	98	114	72	104	105	100	112	72	102	107	0	0	0	0	1	28	7.90	0
407	1	11	156	50	20.55	87	100	64	94	96	95	116	88	98	98	92	112	84	105	96	103	103	57	97	105	1	0	0	0	0	18	8.70	0
408	1	12	161	63	24.30	82	135	86	86	91	87	140	90	91	88	89	137	89	96	89	89	131	74	94	96	0	0	0	0	1	14	8.20	0
409	1	11	142	32	15.87	65	91	60	77	84	82	102	75	94	90	87	97	67	84	92	88	106	56	89	94	1	0	0	1	1	29	6.50	0
410	1	12	158	48	19.23	81	118	70	110	116	108	122	85	104	114	113	122	76	116	109	108	113	83	116	116	0	0	0	0	1	35	8.10	0
411	1	11	158	63	25.24	76	127	56	96	100	94	137	67	103	105	90	135	71	104	102	103	138	77	104	105	1	0	0	0	0	29	7.60	0
412	1	10	140	39	19.90	77	106	56	85	86	92	108	69	93	102	88	105	66	92	92	93	104	73	90	102	0	0	0	0	0	25	7.70	0
413	1	12	152	38	16.45	89	115	63	118	118	117	126	75	119	122	117	122	70	117	120	123	126	72	130	130	0	0	0	1	1	41	8.90	1
414	1	10	146	35	16.42	72	103	60	93	86	91	116	71	89	96	87	109	76	99	91	87	118	72	86	99	0	0	0	0	1	27	7.20	0
415	1	12	160	51	19.92	77	126	65	90	101	94	113	64	90	100	97	119	73	97	105	94	116	67	97	105	1	0	0	1	1	28	7.70	0
416	1	11	148	41	18.72	81	118	50	106	87	90	124	67	94	97	98	110	65	86	92	104	125	63	95	106	0	1	0	0	1	25	8.10	0
417	1	11	146	38	17.83	78	104	73	88	72	81	112	77	84	95	90	113	77	91	88	85	116	79	81	95	0	0	0	1	0	17	7.80	0
419	1	11	152	46	19.91	99	112	69	109	113	109	125	81	113	113	110	122	76	114	116	113	136	76	114	116	1	0	0	0	0	17	9.90	0
421	1	10	143	30	14.67	82	107	61	88	92	96	112	74	97	95	96	109	74	99	93	94	115	73	89	99	0	0	0	0	1	17	8.20	0
422	1	11	152	62	26.84	65	113	67	90	80	90	114	78	86	91	77	109	75	88	75	81	128	77	90	91	1	0	0	1	1	26	6.50	0
424	1	10	143	35	17.12	81	108	73	104	96	94	115	78	89	92	89	113	76	90	87	92	117	73	89	104	0	0	0	0	1	23	8.10	0
426	2	9	143	40	19.56	73	120	76	73	80	84	115	80	76	82	81	131	98	85	78	90	127	88	77	90	0	1	0	0	1	17	7.30	0
427	1	10	141	35	17.60	85	109	66	95	97	99	119	79	101	97	93	119	69	90			116	81		101	1	0	0	0	0	16	8.50	0
428	2	10	141	37	18.61	74	117	74	92	95	88	119	91	96	92	92	111	83	100	96	86	124	80	91	100	1	1	0	0	1	26	7.40	0
429	2	9	137	28	14.92	85	107	71	93	95	92	110	83	93	92	84	108	81	95	96	95	110	79	94	96	1	0	0	0	1	11	8.50	0
430	2	10	141	39	19.62	83	104	56	105	116	110	126	86	115	127	117	125	88	117	115	121	126	85	122	127	1	0	0	1	0	44	8.30	1
431	2	10	143	43	21.03	92	122	41	96	102	106	121	61	104	105	110	127	63	100	99	104	125	61	104	110	1	1	0	0	0	18	9.20	0
432	2	10	138	37	19.43	80	107	68	97	102	103	123	78	98	102	103	127	85	99	94	97	128	81	100	103	1	0	0	0	0	23	8.00	0
433	2	11	156	56	23.01	76	111	68	92	95	98	123	82	104	103	101	121	82	100	95	98	118	82	100	104	1	0	0	1	1	28	7.60	0
434	2	10	143	30	14.67	78	118	42	107	102	101	120	65	102	106	96	101	68	101	110	97	105	72	106	110	1	0	0	0	1	32	7.80	0

Test group

code	sex	age	height	weight	BMI	supine HR	supine SBP	supine DBP	HR1	HR2	HR3	SBP3	DBP3	HR4	HR5	HR6	SBP6	DBP6	HR7	HR8	HR9	SBP9	DBP9	HR10	highest HR	car sick	family history	water intake	sleeping hours	school-indiced burden	HR increase	supine HR10	POTS
435	2	9	137	30	15.98	74	103	61	81	89	96	118	72	96	100	97	123	84	87	99	98	110	78	87	100	1	0	0	0	1	26	7.40	0
436	1	10	141	37	18.61	75	103	62	88	86	82	113	78	80	91	85	119	70	92	91	82	112	78	89	92	0	0	0	0	1	17	7.50	0
437	2	10	125	25	16.00	87	111	62	92	95	96	127	82	95	100	105	124	77	102	110	92	124	83	103	110	1	1	0	0	0	23	8.70	0
438	2	10	139	28	14.49	85	111	67	101	96	91	118	70	102	101	101	115	78	107	107	99	111	83	106	107	1	1	0	0	1	22	8.50	0
439	2	10	140	33	16.84	115	139	80	122	122	128	144	96	122	123	128	159	93	121	125	126	142	88	125	128	0	0	0	0	0	13	11.50	0
440	2	10	141	44	22.13	88	109	48	93	94	102	117	69	106	104	101	111	72	111	107	113	109	71	105	113	1	0	0	0	1	25	8.80	0
441	1	10	145	39	18.55	102	110	70	120	124	125	122	80	121	120	120	118	81	125	119	118	116	76	121	125	0	0	0	0	0	23	10.20	0
442	1	8	140	45	22.96	72	116	57	78	75	74	115	60	81	74	77	116	54	79	77	81	118	56	79	81	1	0	0	0	0	9	7.20	0
444	1	10	131	34	19.81	87	103	65	102	102	105	115	78	101	99	103	114	78	104	107	99	117	80	106	107	1	0	0	0	1	20	8.70	0
445	1	10	145	35	16.65	92	108	65	96	97	101	113	74	98	97	103	107	72	103	96		110	73		103	0	0	0	0	0	11	9.20	0
446	1	10	137	35	18.65	86	94	45	106	105	106	102	68	105	112	110	105	69	113	109	105	97	60	109	113	0	1	0	0	1	27	8.60	0
448	1	10	142	36	17.85	96	108	62	105	106	107	88	67	109	110	105	103	52	109	117	114	110	73	103	117	1	0	0	0	0	21	9.60	0
450	1	10	148	36	16.44	100	127	63	113	114	117	121	70	118	116	114	125	76	117	109	112	129	81	112	118	0	0	0	0	0	18	10.00	0
451	2	9	143	35	17.12	90	124	71	97	100	101	121	77	95	103	105	119	83	97	103	100	113	77	98	105	1	0	0	0	0	15	9.00	0
452	2	10	140	30	15.31	90	110	58	116	105	112	123	64	107	94	98	121	62	102	101	96	122	51	107	116	1	0	0	1	1	26	9.00	0
453	2	11	145	46	21.88	96	117	82	110	104	107	130	87	113	108	103	133	81	108	111	105	138	77	93	113	0	0	0	0	1	17	9.60	0
454	2	10	140	31	15.82	97	107	69	105	106	105	115	79	107	108	100	107	72	104	112	106	113	46	109	112	0	0	0	0	1	15	9.70	0
455	2	10	150	57	25.33	93	113	63	96	113	115	124	71	104	109	113	123	71	115	110	106	126	73	106	115	0	0	0	0	0	22	9.30	0
456	2	11	149	39	17.57	82	103	60	93	100	101	114	76	103	100	103	114	82	95	97	98	121	80	100	103	0	1	0	0	1	21	8.20	0
458	1	11	145	35	16.65	85	102	43	101	102	99	112	72	96	98	96	120	62	98	100	97	110	67	95	102	0	0	0	0	1	17	8.50	0
459	2	10	141	35	17.60	105	114	71	112	112	116	126	82	112	120	123	126	79	125	120	118	126	88	126	126	1	0	1	1	1	21	10.50	0
460	1	10	146	40	18.77	93	103	70	99	95	89	119	66	99	92	100	131	76	92	94	97	127	79	103	103	0	0	0	1	1	10	9.30	0
461	2	10	145	39	18.55	72	117	55	84	93	85	127	77	94	96	90	120	71	93	97	91	129	76	95	97	0	0	0	0	0	25	7.20	0
462	1	11	160	63	24.61	105	147	71	122	120	110	145	97	107	111	108	148	87	104	111	105	161	83	104	122	1	0	0	0	0	17	10.50	0
464	2	10	155	40	16.65	84	112	77	107	108	104	119	83	105	116	112	116	79	106	110	107	114	79	106	116	0	0	0	0	0	32	8.40	0
465	2	10	154	56	23.61	81	121	72	120	107	111	127	84	110	107	106	128	81	110	108	110	127	89	106	120	1	0	0	0	1	39	8.10	0
466	1	11	144	29	13.99	94	109	77	101	102	109	123	93	105	106	108	114	80	111	103	113	119	86	101	113	0	0	0	0	1	19	9.40	0
468	2	10	151	29	12.72	100	105	68	112	113	98	115	69	114	105	101	120	79	105	104	108	114	74	107	114	1	1	0	0	0	14	10.00	0
469	1	11	151	49	21.49	97	124	60	108	115	118	132	86	113	111	110	127	88	116	108	108	126	86	118	118	0	0	0	1	1	21	9.70	0
470	2	10	149	26	11.71	93	99	64	104	102	108	110	78	110	113	108	110	74	111	114	113	104	66	112	114	1	0	0	0	0	21	9.30	0
471	2	10	152	67	29.00	100	114	53	98	98	110	149	128	105	124	90	164	143	119	112	111	141	60	115	124	1	0	0	0	1	24	10.00	0
472	1	11	153	42	17.94	95	112	69	99	100	105	130	88	103	100	98	121	86	113	107	109	121	76	102	113	1	0	0	0	1	18	9.50	0
473	2	10	138	36	18.90	85	108	70	97	98	104	103	67	113	108	97	104	70	116	97	103	110	72	104	116	0	0	0	0	0	31	8.50	0
474	2	12	131	35	20.40	98	111	71	121	107	122	124	64	111	107	112	114	82	112	110	112	120	80	114	122	1	0	0	1	1	24	9.80	0
475	1	10	155	58	24.14	84	110	63	110	109	109	111	74	110	111	110	108	66	112	111	115	108	73	114	115	1	0	0	0	1	31	8.40	0
477	2	10	137	27	14.39	96	97	47	98	112	115	107	66	114	112	120	114	62	114	117	118	116	67	114	120	1	0	1	1	1	24	9.60	1
478	1	11	140	31	15.82	99	116	68	117	102	105	127	79	101	110	103	124	78	100	103	107	115	81	110	117	0	0	0	1	1	18	9.90	0
479	1	11	148	39	17.80	86	113	55	98	100	108	121	71	105	101	104	114	61	101	103	104	116	61	108	108	0	0	0	0	0	22	8.60	0
480	1	11	165	50	18.37	98	117	64	109	108	108	124	82	102	110	110	126	81	113	106	107	126	80	110	113	0	1	0	0	0	15	9.80	0
481	1	10	157	43	17.44	86	123	61	115	114	114	124	85	111	114	112	126	88	111	114	111	127	82	115	115	1	0	0	0	0	29	8.60	0
483	1	11	140	36	18.37	72	104	57	100	96	92	118	61	93	98	90	121	60	97	98	93	115	71	99	100	0	0	1	0	1	28	7.20	0
484	1	12	153	50	21.36	82	102	54	112	110	111	115	65	113	116	112	109	69	112	111	112	112	67	118	118	0	0	0	0	0	36	8.20	0
485	1	12	146	40	18.77	77	112	59	86	87	86	112	75	90	91	92	117	70	95	92	90	110	77	91	95	1	0	0	0	1	18	7.70	0
486	1	11	165	53	19.47	88	112	53	108	110	116	122	74	106	113	106	109	66	109	109	106	113	73	115	116	0	0	0	0	1	28	8.80	0
487	1	11	144	34	16.40	80	98	65	85	91	91	103	70	90	91	93	110	66	100	95	98	108	72	96	100	1	0	0	0	1	20	8.00	0
488	1	10	137	39	20.78	86	101	73	106	102	105	115	74	110	101	94	118	73	103	107	106	116	77	102	110	0	0	0	0	1	24	8.60	0
489	1	11	154	59	24.88	87	107	65	100	103	96	123	75	103	97	98	114	76	100	110	98	117	76	107	110	1	0	0	0	0	23	8.70	0
490	1	10	134	31	17.26	79	101	61	118	101	98	111	69	102	98	96	115	63	104	105	108	114	58	95	118	1	0	0	0	0	39	7.90	0
492	1	12	164	46	17.10	96	107	57	113	117	113	126	79	112	118	119	127	75	122	115	118	127	83	125	125	0	0	0	0	1	29	9.60	0
493	1	10	133	28	15.83	104	110	66	119	107	105	107	80	106	108	104	108	80	107	104	106	112	77	108	119	0	1	0	0	0	15	10.40	0
494	1	10	139	32	16.56	79	111	46	80	92	87	112	69	91	92	96	109	65	95	95	87	104	67	90	96	0	0	0	0	1	17	7.90	0
496	2	11	155	39	16.23	92	120	72	109	98	97	118	83	93	102	98	118	82	107	101	106	121	73	100	109	1	0	0	0	1	17	9.20	0
497	2	9	142	24	11.90	92	113	66	100	109	107	130	78	116	107	113	127	77	113	107	116	127	72	114	116	0	1	0	0	0	24	9.20	0
498	1	10	136	37	20.00	72	96	59	82	84	84	105	67	86	91	84	108	77	85	88	83	108	77	87	91	0	1	0	0	1	19	7.20	0
499	1	11	150	45	20.00	80	91	50	111	108	104	109	74	97	103	98	108	59	104	108	103	89	58	96	111	0	0	0	0	1	31	8.00	0

Test group

code	sex	age	height	weight	BMI	supine HR	supine SBP	supine DBP	HR1	HR2	HR3	SBP3	DBP3	HR4	HR5	HR6	SBP6	DBP6	HR7	HR8	HR9	SBP9	DBP9	HR10	highest HR	car sick	family history	water intake	sleeping hours	school-indiced burden	HR increase	supine HR10	POTS
502	1	11	156	40	16.44	78	108	52	93	90	89	118	67	91	90	89	113	65	89	88	84	111	65	83	93	0	0	0	0	0	15	7.80	0
505	1	11	153	46	19.65	88	106	62	109	107	99	116	71	98	100	97	118	77	103	110	105	116	76	112	112	1	0	0	0	1	24	8.80	0
506	1	12	157	44	17.85	88	106	57	104	104	107	114	76	111	111	108	115	72	110	108	106	119	60	116	116	0	0	0	0	0	28	8.80	0
507	1	11	150	44	19.56	78	111	53	93	93	94	119	70	100	88	92	119	76	90	95	88	119	66	93	100	1	0	0	0	1	22	7.80	0
508	1	12	144	32	15.43	85	104	63	98	100	106	112	78	96	101	107	113	76	105	106	105	108	69	111	111	1	0	0	0	1	26	8.50	0
509	1	11	150	49	21.78	80	113	67	106	107	101	125	81	100	102	98	111	83	100	99	98	119	78	99	107	1	0	0	0	1	27	8.00	0
511	2	11	154	53	22.35	79	111	65	102	96	101	128	65	94	103	90	114	69	102	96	89	127	71	95	102	1	0	0	0	1	23	7.90	0
512	2	11	161	59	22.76	92	107	60	110	103	104	127	84	110	109	98	134	69	113	108	107	123	81	110	113	1	0	0	0	1	21	9.20	0
513	2	12	150	39	17.33	89	112	76	99	97	102	116	83	104	100	98	112	77	103	108	107	110	81	100	108	0	0	0	0	1	19	8.90	0
514	2	11	161	77	29.71	73	106	67	83	75	75	118	57	79	79	75	120	76	87	87	84	99	74	82	87	1	0	0	0	1	14	7.30	0
515	2	10	153	51	21.79	81	128	77	101	94	92	137	73	95	99	98	156	84	109	101	97	153	120	105	109	1	0	0	0	1	28	8.10	0
516	2	10	150	38	16.89	89	104	45	110	103	96	86	58	110	104	98	105	49	105	104	109	112	44	105	110	1	0	0	0	1	21	8.90	0
517	2	11	146	33	15.48	97	102	58	117	107	120	110	76	117	115	119	107	73	118	123	114	106	74	120	123	0	0	1	1	1	26	9.70	1
518	2	10	159	49	19.38	89	99	59	105	108	108	107	68	99	103	107	112	66	103	105	108	108	70	108	108	0	0	0	0	1	19	8.90	0
519	2	10	146	34	15.95	105	103	62	120	125	120	113	77	129	117	121	115	80	126	119	123	130	88	134	134	0	0	0	0	0	29	10.50	0
521	2	10	148	45	20.54	96	113	58	119	113	114	115	73	116	121	118	112	74	121	121	117	117	78	118	121	1	0	0	1	1	25	9.60	0
523	2	11	154	49	20.66	93	111	68	106	107	109	121	78	108	100	111	119	77	106	110	113	118	80	100	113	1	0	0	0	1	20	9.30	0
524	2	12	148	58	26.48	102	117	69	126	116	117	121	78	127	133	122	91	66	128	125	128	129	81	123	133	1	0	0	1	1	31	10.20	1
525	2	11	144	34	16.40	75	92	59	92	102	97	108	66	113	118	104	95	63	102	106	99	107	69	107	118	1	0	1	1	1	43	7.50	1
526	2	12	161	64	24.69	78	139	66	94	92	98	123	73	101	94	98	127	83	95	98	102	129	82	97	102	0	0	0	1	0	24	7.80	0
527	2	11	156	44	18.08	80	99	60	90	90	85	103	70	89	85	86	107	64	83	84	93	108	64	91	93	1	1	0	1	1	13	8.00	0
530	2	11	150	51	22.67	98	101	45	119	110	110	114	66	114	114	111	122	56	110	112	109	113	57	118	119	1	0	0	1	1	21	9.80	0
531	2	11	157	46	18.66	97	101	65	124	113	117	109	80	111	121	112	120	78	119	127	127	114	71	118	127	1	0	0	0	1	30	9.70	1
532	2	10	137	30	15.98	84	97	64	93	82	92	106	73	93	95	95	112	73	92	92	92	108	73	89	95	1	1	0	0	0	11	8.40	0
536	2	10	148	43	19.63	85	127	80	112	98	87	134	88	99	91	101	127	87	113	102	99	130	93	95	113	1	0	0	0	1	28	8.50	0
537	2	11	145	37	17.60	92	111	71	116	101	106	113	71	104	101	97	106	71	101	107	101	111	73	103	116	0	1	0	1	1	24	9.20	0
538	2	11	143	36	17.60	87	107	51	112	108	108	121	76	113	113	106	121	59	110	111	109	124	73	110	113	1	0	0	0	1	26	8.70	0
541	2	10	148	41	18.72	80	104	65	94	98	97	116	80	91	96	95	118	79	96	96	104	117	78	97	104	1	0	0	0	1	24	8.00	0
543	2	11	161	63	24.30	90	113	72	89	92	92	125	91	97	96	96	124	88	95	100	93	119	87	96	100	1	0	0	0	1	10	9.00	0
545	1	11	150	42	18.67	88	127	57	101	104	107	123	64	109	105	109	122	66	112	107	108	124	60	112	112	0	0	0	1	0	24	8.80	0
549	2	11	158	70	28.04	72	110	66	75	84	84	128	87	81	90	86	125	80	86	75	88	121	81	89	90	1	0	0	0	1	18	7.20	0
550	2	10	130	27	15.98	84	109	70	105	108	102	116	81	112	114	107	113	74	104	108	111	112	74	101	114	1	0	0	0	0	30	8.40	0
552	2	11	160	47	18.36	95	133	76	116	111	103	142	85	117	109	107	138	91	105	111	98	136	92	105	117	0	0	0	0	1	22	9.50	0
553	2	12	157	43	17.44	83	119	70	106	106	100	130	70	105	101	108	126	64	101	102	107	124	73	102	108	0	0	0	1	1	25	8.30	0
554	1	11	153	35	14.95	75	114	76	93	83	91	131	89	89	89	89	121	86	91	89	90	127	85	90	93	0	0	0	0	0	18	7.50	0
556	1	11	153	42	17.94	78	118	62	86	89	90	115	77	89	91	90	114	75	93	91	90	118	74	89	93	1	0	0	0	1	15	7.80	0
560	1	11	156	70	28.76	78	113	48	88	94	93	115	73	94	99	98	168	144	98	93	96	111	57	93	99	1	0	0	1	1	21	7.80	0
562	2	12	158	48	19.23	84	115	60	92	108	105	123	73	100	109	103	133	69	110	115	116	121	71	107	116	1	0	0	0	1	32	8.40	0
564	2	12	158	49	19.63	76	109	65	100	104	100	120	84	107	100	101	116	72	107	104	98	126	82	103	107	1	1	0	0	1	31	7.60	0
565	2	10	143	35	17.12	87	94	53	94	85	93	104	74	95	92	98	105	67	94	100	95	107	71	94	100	0	0	0	0	0	13	8.70	0
568	2	10	130	29	17.16	83	83	50	102	105	104	94	69	102	98	99	104	67	110	96	107	99	63	110	110	1	0	0	0	1	27	8.30	0
569	2	10	135	26	14.27	114	119	56	135	124	121	109	75	132	129	130	124	78	134	128	130	127	74	134	135	1	0	0	0	0	21	11.40	0
571	1	11	137	33	17.58	88	115	63	105	105	105	129	97	108	100	108	131	83	109	110	112	125	87	106	112	0	0	0	0	0	24	8.80	0
572	2	9	131	25	14.57	87	104	55	110	99	93	112	72	107	92	100	109	65	104	91	101	110	74	105	110	1	0	0	0	0	23	8.70	0
573	1	11	157	45	18.26	77	112	66	86	88	90	112	69	90	95	96	119	71	100	93	96	112	75	104	104	0	0	0	0	0	27	7.70	0
574	1	11	154	40	16.87	70	95	51	88	87	85	105	57	88	89	88	105	50	91	91	92	99	53	85	91	1	0	0	0	1	21	7.00	0
575	1	11	141	32	16.10	87	111	73	99	101	108	121	85	106	103	102	124	89	107	111	109	128	87	114	114	0	0	0	0	1	27	8.70	0
576	1	13	152	42	18.18	66	109	62	76	80	80	111	74	90	82	79	118	76	87	93	82	113	75	80	93	0	0	0	0	1	27	6.60	0
577	1	10	141	32	16.10	78	98	56	108	110	109	103	80	115	111	114	112	76	108	115	108	110	73	111	115	0	0	0	0	1	37	7.80	0
578	1	11	146	42	19.70	89	109	61	114	119	118	114	87	121	116	112	105	61	117	124	120	110	63	121	124	1	1	0	1	1	35	8.90	0
579	2	11	163	63	23.71	88	102	63	103	89	95	120	82	96	89	90	135	71	98	97	98	121	70	97	103	1	0	0	1	1	15	8.80	0
581	1	12	155	54	22.48	78	106	48	95	89	88	112	60	96	96	93	113	62	99	97	95	112	58	90	99	0	0	0	0	1	21	7.80	0
584	2	12	164	47	17.47	93	118	64	118	121	123	130	78	126	126	128	122	80	117	124	122	120	75	121	128	0	0	0	0	1	35	9.30	0
587	1	10	140	29	14.80	89	100	59	101	107	105	115	67	108	109	106	116	67	111	107	106	113	69	108	111	1	0	0	0	1	22	8.90	0
588	2	10	142	31	15.37	88	106	56	111	105	104	119	71	105	105	106	117	58	112	110	107	114	72	110	112	1	0	0	0	1	24	8.80	0

Test group

code	sex	age	height	weight	BMI	supine HR	supine SBP	supine DBP	HR1	HR2	HR3	SBP3	DBP3	HR4	HR5	HR6	SBP6	DBP6	HR7	HR8	HR9	SBP9	DBP9	HR10	highest HR	car sick	family history	water intake	sleeping hours	school-indiced burden	HR increase	supine HR10	POTS
589	2	10	148	36	16.44	93	93	60	117	122	116	108	71	123	121	111	114	74	121	119	113	113	53	119	123	0	1	0	0	1	30	9.30	0
591	1	10	151	41	17.98	80	112	58	102	94	103	120	75	104	107	98	120	75	108	106	110	119	64	119	119	0	0	0	0	0	39	8.00	0
593	2	11	155	45	18.73	72	96	51	78	77	78	97	56	76	77	82	97	57	78	76	79	96	53	78	82	1	1	0	1	1	10	7.20	0
594	2	11	155	47	19.56	73	115	69	95	94	92	132	72	90	95	93	119	86	91	93	91	127	79	97	97	1	1	0	0	1	24	7.30	0
595	2	12	162	55	20.96	69	120	67	83	89	81	113	79	83	86	83	117	66	89	84	82	117	78	77	89	0	0	0	0	0	20	6.90	0
596	1	10	136	40	21.63	81	126	59	102	105	108	114	65	102	108	103	106	65	105	101	104	111	64	108	108	0	0	0	0	0	27	8.10	0
597	1	11	150	43	19.11	77	131	44	98	87	93	124	68	88	87	89	137	55	102	100	96	132	48	100	102	0	0	0	0	1	25	7.70	0
599	1	11	168	65	23.03	93	114	60	126	116	111	112	72	113	119	119	117	75	121	125	115	97	67	112	126	1	0	0	1	1	33	9.30	1
604	1	10	143	37	18.09	86	101	59	104	104	103	107	77	106	105	113	101	63	113	104	102	110	72	112	113	1	0	0	0	1	27	8.60	0
606	1	10	144	31	14.95	70	105	65	81	75	71	117	76	76	74	71	117	71	67	66	72	115	77	73	81	0	0	0	0	1	11	7.00	0
607	1	11	153	57	24.35	83	125	83	110	98	99	134	95	105	103	93	128	96	97	105	113	132	95	108	113	1	0	0	0	1	30	8.30	0
608	2	10	154	31	13.07	88	114	73	104	101	109	127	86	114	104	107	124	90	109	101	110	126	85	112	114	1	0	0	0	1	26	8.80	0
610	1	10	148	47	21.46	104	135	51	117	110	116	121	67	113	119	120	121	64	116	118	120	109	54	117	120	0	0	1	0	1	16	10.40	1
611	1	11	148	43	19.63	79	113	64	106	101	103	107	73	108	111	105	106	72	110	107	107	109	65	110	111	1	0	0	0	1	32	7.90	0
614	1	11	153	36	15.38	84	100	74	110	114	117	111	72	114	113	117	102	68	115	113	115	117	78	112	117	0	0	0	1	1	33	8.40	0
615	2	10	142	31	15.37	79	100	63	94	97	96	120	57	98	101	102	108	59	100	99	95	104	64	103	103	1	0	0	0	1	24	7.90	0
616	2	10	149	47	21.17	80	118	73	108	106	97	136	91	105	117	111	131	83	112	115	108	129	87	109	117	1	0	0	0	1	37	8.00	0
617	1	12	164	50	18.59	79	117	96	98	102	106	121	69	100	104	106	138	62	107			105	55		107	1	0	0	1	0	28	7.90	0
618	2	11	151	52	22.81	91	136	73	111	110	105	141	76	100	107	114	150	78	109	104	111	118	76	106	111	1	0	0	0	1	20	9.10	0
619	2	11	156	46	18.90	74	102	60	94	92	90	117	68	101	94	95	107	64	89	100	89	109	67	88	101	1	0	0	0	1	27	7.40	0
620	1	12	156	60	24.65	85	122	51	110	102	97	117	73	106	107	100	130	69	109	106	108	74	56	107	110	0	0	0	0	0	25	8.50	0
621	2	12	153	47	20.08	80	108	64	107	118	111	114	78	122	118	114	113	72	115	113	120	115	70	106	122	1	0	0	1	1	42	8.00	1
623	1	11	151	39	17.10	83	131	85	103	95	101	127	78	98	100	98	117	82	103	106	104	119	80	111	111	0	0	0	0	0	28	8.30	0
624	2	10	155	46	19.15	107	114	77	124	106	110	122	90	109	120	114	116	84	107	116	118	120	85	111	124	1	0	0	1	1	17	10.70	1
625	2	10	136	28	15.14	110	123	77	122	115	116	134	91	113	119	122	130	87	122	123	123	125	85	118	123	0	0	0	0	0	13	11.00	0
627	1	11	150	40	17.78	85	105	74	102	112	109	109	80	103	109	116	116	73	109	100	114	110	79	109	116	0	0	0	0	1	31	8.50	0
628	2	10	154	40	16.87	106	115	77	121	113	110	116	77	112	108	109	117	74	115	116	114	114	80	118	121	0	0	0	0	1	15	10.60	0
630	2	11	160	56	21.88	76	123	61	101	87	92	125	69	96	93	90	126	58	98	94	93	130	69	90	101	1	0	0	0	1	25	7.60	0
704	2	12	153	44	18.80	73	98	56	90	85	83	117	80	89	93	93	116	74	95	84	92	115	77	88	95	0	1	0	0	0	22	7.30	0
705	1	12	143	32	15.65	71	91	55	74	79	77	101	65	79	86	77	98	65	75	81	95	104	73	74	95	1	0	0	0	1	24	7.10	0
722	2	9	145	32	15.22	84	98	54	96	92	95	102	72	92	92	96	108	73	102	96	94	105	66	98	102	1	0	0	0	0	18	8.40	0
728	2	13	156	48	19.72	74	112	68	96	101	93	123	70	101	102	104	123	77	101	102	101	115	68	100	104	1	0	0	0	0	30	7.40	0
730	2	9	130	30	17.75	84	101	58	87	94	96	102	76	103	108	93	100	70	101	99	104	96	58	102	108	0	0	0	0	0	24	8.40	0
731	2	9	144	42	20.25	87	115	63	102	96	103	126	80	96	101	104	121	73	103	103	103	115	76	103	104	0	0	0	0	1	17	8.70	0
746	2	14	156	52	21.37	110	112	61	118	118	117	116	71	124	122	119	114	74	128	127	127	111	75	124	128	0	0	0	0	1	18	11.00	0
754	2	12	147	35	16.20	83	89	47	106	105	99	111	76	97	109	107	10	9	98	107	106	107	6	105	109	1	1	0	1	1	26	8.30	0
782	1	9	144	39	18.81	102	108	64	117	105	116	120	82	112	115	111	121	76	115	116	110	124	77	105	117	1	0	0	0	0	15	10.20	0
785	1	9	133	29	16.39	90	101	61	105	99	91	99	73	111	101	107	113	69	104	106	102	106	57	95	107	1	1	0	0	0	17	9.00	0
807	1	8	130	24	14.20	75	91	56	103	97	94	103	75	102	95	90	112	80	101	93	91	107	77	96	103	1	0	0	0	1	28	7.50	0
816	1	11	146	30	14.07	96	111	72	118	110	110	120	88	108	105	105	122	85	106	106	107	120	83	108	118	0	0	0	0	1	22	9.60	0
820	2	11	138	30	15.75	80	118	62	90	96	91	127	77	100	101	104	117	75	97	102	102	121	73	97	104	0	0	0	0	1	24	8.00	0
823	1	11	138	32	16.80	78	101	62	83	96	103	113	81	95	97	99	110	81	96	95	96	122	94	94	103	1	0	0	1	0	25	7.80	0
832	1	8	145	27	12.84	76	107	57	112	110	107	110	63	113	110	107	103	65	108	115	103	110	70	110	115	1	0	0	0	0	39	7.60	0
835	1	8	152	50	21.64	88	115	68	117	102	98	120	82	104	101	102	124	81	112	111	108	118	65	110	117	0	0	0	0	0	29	8.80	0
838	1	8	145	26	12.37	94	104	66	116	106	100	109	81	111	110	108	108	80	107	110	101	111	82	108	116	0	0	0	0	0	22	9.40	0
843	1	11	146	48	22.52	91	114	70	110	104	108	129	85	108	105	106	122	79	106	104	106	124	82	110	110	0	0	0	0	0	19	9.10	0
850	2	11	141	35	17.60	101	102	57	117	111	113	107	65	115	113	114	111	67	117	119	113	107	64	117	119	1	0	0	0	1	18	10.10	0
856	1	8	135	27	14.81	82	103	51	106	112	114	111	66	123			103	59							123	1	0	1	0	0	41	8.20	1
857	2	8	135	27	14.81	78	115	67	89	93	96	118	80	93	95	84	110	71	103	95	90	113	75	95	103	1	0	0	0	1	25	7.80	0
858	1	8	143	33	16.14	108	113	62	138	138	127	123	80	136	142	138	122	79	135	133	133	116	76	127	142	1	0	0	0	1	34	10.80	0
859	1	8	121	23	15.71	79	92	63	103	93	101	96	66	94	100	95	97	71	93	92	91	102	72	94	103	1	0	0	0	0	24	7.90	0
867	2	8	125	25	16.00	89	111	63	104	105	103	118	77	104	109	107	117	70	108	112	102	117	70	110	112	0	0	0	0	1	23	8.90	0
870	2	8	132	27	15.50	79	97	60	97	94	99	98	66	92	103	95	103	71	96	98	100	107	67	99	103	1	0	0	0	1	24	7.90	0
871	1	8	134	27	15.04	88	98	65	112	108	110	116	77	112	116	112	114	79	117	115	117	114	81	115	117	0	0	0	0	1	29	8.80	0
872	1	11	125	28	17.92	78	98	64	88	97	95	102	74	96	95	91	106	77	98	96	100	109	77	97	100	1	0	0	1	1	22	7.80	0

Test group

code	sex	age	height	weight	BMI	supine HR	supine SBP	supine DBP	HR1	HR2	HR3	SBP3	DBP3	HR4	HR5	HR6	SBP6	DBP6	HR7	HR8	HR9	SBP9	DBP9	HR10	highest HR	car sick	family history	water intake	sleeping hours	school-indiced burden	HR increase	supine HR10	POTS
887	1	8	124	26	16.91	96	109	73	104	107	107	123	91	104	106	108	116	82	106	109	108	122	86	106	109	0	0	0	0	1	13	9.60	0
892	1	8	134	27	15.04	83	111	74	90	94	88	121	88	85	89	94	117	84	99	90	92	112	84	93	99	0	0	0	0	1	16	8.30	0
894	2	8	120	22	15.28	110	92	50	126	120	121	102	62	126	125	123	100	65	118	126	119	102	63	126	126	1	1	1	0	1	16	11.00	0
901	1	8	132	29	16.64	81	89	56	102	103	94	96	69	96	96	100	93	67	101	110	98	95	66	100	110	1	0	0	0	1	29	8.10	0
906	1	8	125	28	17.92	97	106	58	113	113	110	114	72	110	115	120	109	68	115	118	120	109	74	122	122	1	0	1	1	0	25	9.70	0
907	2	8	125	24	15.36	95	95	63	110	111	106	103	60	108	110	111	101	65	110	109	109	108	69	111	111	0	1	0	0	0	16	9.50	0
915	1	12	153	50	21.36	90	105	63	117	116	111	114	75	117	112	118	113	74	120	116	118	113	76	120	120	1	0	0	0	0	30	9.00	0
919	1	8	150	40	17.78	83	100	66	108	108	110	113	74	115	114	122	114	78	120	118	117	116	79	124	124	1	0	1	0	0	41	8.30	1
922	1	9	137	26	13.85	94	97	65	124	108	115	111	77	116	120	119	110	87	113	123	120	96	60	120	124	0	0	1	1	0	30	9.40	1
924	1	8	120	22	15.28	111	99	58	144	133	129	112	79	129	129	131	107	76	121	127	128	107	73	132	144	0	0	0	0	1	33	11.10	0
925	2	8	129	25	15.02	82	106	75	106	104	102	114	79	102	108	98	115	84	106	107	107	104	74	105	108	0	0	0	0	0	26	8.20	0
926	2	8	125	23	14.72	70	110	59	80	93	82	115	75	91	85	86	111	78	90	89	91	107	72	86	93	1	0	0	0	1	23	7.00	0
930	1	7	125	25	16.00	121	115	67	128	126	128	123	83	126	128	133	116	80	129	131	129	122	71	127	133	0	0	1	1	0	12	12.10	0
931	2	8	132	30	17.22	80	114	64	108	100	105	121	75	110	107	103	120	70	110	110	108	153	131	102	110	0	0	1	1	0	30	8.00	0
932	1	12	134	27	15.04	85	86	62	103	101	101	109	82	111	105	108	107	80	108	103	108	107	78	105	111	1	0	0	1	0	26	8.50	0
933	1	8	134	28	15.59	70	96	50	74	83	83	105	66	76	97	93	103	73	85	90	91	105	68	85	97	0	0	0	0	0	27	7.00	0
934	2	9	122	22	14.78	96	111	64	105	110	104	119	67	102	105	107	120	75	103	103	108	112	72	103	110	0	0	0	0	0	14	9.60	0
935	1	8	125	25	16.00	80	99	58	99	108	102	99	71	94	95	100	97	69	101	98	104	105	71	101	108	0	0	0	0	1	28	8.00	0
937	1	8	125	27	17.28	86	99	62	101	86	90	114	76	91	94	97	113	77	99	95	99	112	80	90	101	1	0	0	0	1	15	8.60	0
938	1	9	121	22	15.03	98	105	65	128	122	114	110	72	125	131	131	109	75	130	125	128	104	76	127	131	1	0	0	0	1	33	9.80	0
941	1	8	125	25	16.00	71	84	46	100	92	89	89	70	97	101	92	104	74	97	96	88	91	59	102	102	0	0	0	0	1	31	7.10	0
943	2	8	125	27	17.28	84	81	45	89	99	98	102	50	94	99	97	103	67	95	96	99	111	60	97	99	1	0	0	0	1	15	8.40	0
945	1	11	142	30	14.88	95	113	66	116	111	109	125	79	114	116	114	124	84	114	112	113	129	83	112	116	1	0	1	0	1	21	9.50	0
954	1	9	140	40	20.41	90	109	54	102	109	103	113	71	104	103	115	117	76	106	102	102	118	70	106	115	1	0	0	0	0	25	9.00	0
955	1	9	132	30	17.22	98	106	66	117	110	116	114	77	119	114	115	114	78	116	115	117	116	78	119	119	1	1	0	1	1	21	9.80	0
956	1	10	142	37	18.35	97	102	42	108	110	108	114	52	106	105	108	106	77	111	109	108	110	63	111	111	0	0	1	0	1	14	9.70	0
957	1	9	132	28	16.07	80	107	65	99	108	102	119	77	102	103	103	117	73	107	105	108	113	67	108	108	0	0	0	0	1	28	8.00	0
959	1	9	134	30	16.71	104	108	75	134	138	142	115	82	134	144	138	122	87	140	142	140	132	89	141	144	0	0	0	1	0	40	10.40	1
960	2	8	123	23	15.20	96	92	54	111	110	113	105	70	110	117	110	108	62	117	114	114	103	66	113	117	1	0	0	0	0	21	9.60	0
961	2	9	131	31	18.06	101	117	45	112	117	120	103	70	119	114	112	119	81	113	116	119	122	73	120	120	0	0	0	0	1	19	10.10	0
962	1	8	132	25	14.35	110	111	70	133	121	127	113	75	126	130	127	121	68	129	127	129	118	68	126	133	0	1	0	0	0	23	11.00	0
964	1	7	117	22	16.07	90	96	62	112	110	108	108	75	112	110	107	115	78	107	102	110	111	78	110	112	0	0	0	0	1	22	9.00	0
965	1	8	135	30	16.46	102	104	56	112	120	121	119	75	115	119	117	116	72	126	122	122	115	77	118	126	1	0	0	0	1	24	10.20	0
966	1	7	125	27	17.28	85	103	58	103	105	109	103	72	109	109	107	103	66	111	112	109	100	67	110	112	1	0	0	0	1	27	8.50	0
967	1	10	155	45	18.73	84	101	61	107	102	105	116	85	111	104	112	125	83	103	110	106	115	81	112	112	1	0	0	0	1	28	8.40	0
968	2	9	145	34	16.17	84	103	63	111	106	107	117	77	105	108	105	118	73	105	104	109	114	83	103	111	0	0	0	0	0	27	8.40	0
969	1	7	134	27	15.04	83	96	60	105	107	97	105	59	105	105	103	111	71	101	108	108	108	64	106	108	1	0	0	0	0	25	8.30	0
970	1	10	143	39	19.07	103	110	44	108	102	101	114	72	106	106	103	115	77	103	106	106	110	74	108	108	1	1	0	0	1	5	10.30	0
971	2	10	144	39	18.81	83	105	73	98	99	107	113	76	105	106	99	117	74	107	107	111	120	81	108	111	1	0	0	0	1	28	8.30	0
1001	2	7	136	30	16.22	87	120	47	106	104	114	117	65	114	116	111	138	71	112	113	104	121	66	114	116	1	0	0	1	1	29	8.70	0
1002	1	10	131	25	14.57	81	115	67	107	104	108	126	80	109	117	116	136	88	112	119	116	132	86	119	119	1	0	0	0	1	38	8.10	0
1003	2	7	133	39	22.05	84	111	61	101	105	102	116	76	106	101	108	115	70	109	109	103	114	72	107	109	0	0	0	0	1	25	8.40	0
1004	1	7	140	30	15.31	92	103	68	126	105	104	110	80	109	116	112	111	76	119	117	116	110	71	107	126	0	0	1	1	0	34	9.20	0
1006	2	7	136	29	15.68	82	98	59	108	95	100	105	69	95	104	100	105	76	106	105	106	109	74	106	108	0	0	0	0	1	26	8.20	0
1007	2	7	135	29	15.91	92	98	54	118	103	110	104	70	108	114	112	105	67	118	112	110	104	60	111	118	0	1	0	0	0	26	9.20	0
1008	1	7	125	27	17.28	76	95	54	104	94	92	105	69	97	92	89	105	73	87	95	86	106	70	98	104	1	0	0	0	0	28	7.60	0
1009	1	8	135	29	15.91	80	113	62	107	91	105	119	79	107	104	102	120	79	104	103	105	125	81	103	107	0	0	0	0	1	27	8.00	0
1012	2	7	140	39	19.90	95	111	54	100	101	104	109	63	102	111	110	114	65	103	104	107	119	74	102	111	0	0	0	0	0	16	9.50	0
1014	2	7	131	23	13.40	75	95	51	82	80	85	95	68	87	92	87	100	67	87	83	91	98	60	86	92	1	0	0	0	0	17	7.50	0
1015	2	7	134	28	15.59	100	101	52	112	110	108	109	66	107	105	102	107	56	106	113	112	91	66	111	113	0	0	0	0	0	13	10.00	0
1016	1	9	140	30	15.31	64	109	66	88	91	94	117	77	94	100	100	115	74	98	100	98	116	79	100	100	1	0	1	0	1	36	6.40	0
1017	1	7	134	32	17.82	97	118	60	114	120	124	125	78	117	108	105	137	71	108	105	109	127	83	107	124	0	0	1	1	0	27	9.70	0
1018	1	8	136	28	15.14	100	118	62	116	100	106	126	76	98	109	110	132	74	108	109	105	138	71	110	116	0	1	0	0	0	16	10.00	0
1019	1	9	145	47	22.35	71	99	54	99	107	105	112	70	104	106	102	113	71	104	103	98	112	67	99	107	0	0	0	1	0	36	7.10	0
1021	1	7	135	28	15.36	80	118	76	102	99	94	115	73	93	91	97	110	65	101	90	93	109	73	100	102	1	1	1	0	1	22	8.00	0

Test group

code	sex	age	height	weight	BMI	supine HR	supine SBP	supine DBP	HR1	HR2	HR3	SBP3	DBP3	HR4	HR5	HR6	SBP6	DBP6	HR7	HR8	HR9	SBP9	DBP9	HR10	highest HR	car sick	family history	water intake	sleeping hours	school-indiced burden	HR increase	supine HR10	POTS
1022	1	7	142	33	16.37	89	99	65	117	107	108	108	79	114	114	106	104	72	104	118	119	106	75	113	119	1	1	0	1	1	30	8.90	0
1023	1	10	152	55	23.81	80	118	66	93	94	95	127	86	90	95	93	129	80	97	95	100	132	78	95	100	0	0	0	0	0	20	8.00	0
1024	2	8	134	25	13.92	80	98	58	99	98	96	103	71	97	89	91	108	78	86	91	90	116	79	90	99	1	0	0	0	0	19	8.00	0
1025	2	9	147	78	36.10	82	115	68	97	92	98	111	81	91	94	91	108	77	102	101	93	117	78	100	102	1	0	0	1	0	20	8.20	0
1027	1	10	150	37	16.44	95	98	63	117	110	108	112	65	114	118	106	110	67	111	108	118	110	68	116	118	1	1	0	0	1	23	9.50	0
1029	1	9	136	27	14.60	78	107	53	88	79	89	123	66	87	96	93	113	80	88	92	92	112	71	87	96	1	0	0	0	0	18	7.80	0
1030	2	9	135	27	14.81	85	101	56	108	107	100	110	74	100	105	109	108	76	103	106	105	107	69	101	109	0	0	0	0	1	24	8.50	0
1032	2	10	134	26	14.48	74	109	74	113	98	102	120	91	102	98	103	116	82	110	102	112	115	82	103	113	0	0	0	0	0	39	7.40	0
1034	2	9	135	26	14.27	81	94	60	96	98	92	107	62	88	94	96	103	69	94	99	100	98	70	101	101	0	1	0	0	0	20	8.10	0
1035	1	9	135	35	19.20	98	109	70	106	117	109	115	80	119	117	122	113	70	117	109	125	121	83	124	125	0	0	1	0	0	27	9.80	1
1037	1	9	135	28	15.36	72	112	65	96	87	89	121	86	87	83	89	107	81	91	82	90	120	77	87	96	0	0	0	0	0	24	7.20	0
1038	1	9	132	26	14.92	94	105	71	106	108	103	119	79	106	108	105	115	64	107	98	98	118	74	103	108	0	0	0	0	1	14	9.40	0
1040	2	9	155	44	18.31	90	130	78	119	122	123	142	97	125	125	124	135	93	124	128	120	134	91	124	128	1	0	0	0	1	38	9.00	0
1041	2	11	140	31	15.82	102	123	69	105	108	105	131	84	108	111	117	124	80	113	115	123	133	81	120	123	1	0	0	0	1	21	10.20	0
1042	2	9	133	27	15.26	82	99	47	85	96	90	97	70	89	95	92	105	63	93	92	87	105	66	90	96	0	0	0	1	1	14	8.20	0
1045	1	9	140	37	18.88	99	112	55	123	128	124	114	72	125	127	123	59	36	117	126	124	101	61	123	127	1	0	0	0	0	28	9.90	0
1046	1	9	140	37	18.88	80	101	61	93	90	91	104	76	93	89	92	106	79	96	97	93	109	72	94	97	0	0	0	0	0	17	8.00	0
1047	2	10	137	30	15.98	100	100	54	106	104	107	110	71	111	114	111	110	68	107	108	110	107	68	113	114	0	0	0	1	1	14	10.00	0
1048	2	9	136	42	22.71	95	106	60	116	116	112	117	67	113	115	118	123	56	114	124	116	125	66	108	124	0	0	0	0	0	29	9.50	0
1049	2	8	138	31	16.28	84	106	68	109	111	114	118	85	118	116	120	112	76	119	126	132	97	58		132	1	0	1	0	0	48	8.40	1
1050	2	9	140	35	17.86	84	101	45	93	98	100	106	65	96	104	93	110	73	98	94	96	98	46	100	104	1	0	0	0	1	20	8.40	0
1051	1	8	134	29	16.15	96	139	68	108	105	105	131	73	105	106	105	122	74	105	102	103	129	68	113	113	0	0	0	0	1	17	9.60	0
1052	1	9	135	40	21.95	82	115	58	102	105	105	118	83	107	96	97	117	79	107	105	104	89	66	104	107	1	0	0	0	0	25	8.20	0
1053	1	11	135	28	15.36	103	112	54	122	115	112	113	69	117	121	116	109	67	130	122	125	111	70	122	130	0	0	0	0	1	27	10.30	0
1054	1	9	138	35	18.38	100	131	75	111	107	102	146	58	117	105	108	144	86	109	113	119	140	75	115	119	0	0	1	0	1	19	10.00	0
1058	1	10	137	32	17.05	84	110	63	110	105	114	127	90	109	112	118	120	83	115	114	113	119	82	118	118	1	0	0	0	1	34	8.40	0
1060	2	11	133	25	14.13	67	103	66	82	84	80	114	78	98	81	97	113	75	105	89	98	107	74	93	105	0	0	0	0	0	38	6.70	0
1061	1	9	136	27	14.60	113	110	61	126	124	125	114	58	128	127	130	118	63	126	132	125	119	76	126	132	0	0	0	0	1	19	11.30	0
1062	1	9	141	28	14.08	81	95	69	105	104	109	99	74	104	108	106	103	74	102	106	110	110	79	107	110	0	0	0	0	0	29	8.10	0
1064	1	8	127	28	17.36	83	112	60	100	100	104	125	77	104	101	98	121	72	104	102	94	116	73	97	104	1	0	0	0	1	21	8.30	0
1065	2	10	141	41	20.62	80	104	61	85	81	93	110	75	90	89	88	108	70	91	91	93	108	74	90	93	1	0	0	0	1	13	8.00	0
1066	1	9	125	25	16.00	88	108	64	100	108	106	115	80	108	110	109	117	84	113	113	116	118	87	114	116	1	0	1	0	0	28	8.80	0
1067	2	10	143	32	15.65	87	130	52	97	105	101	121	64	104	106	103	130	73	105	110	104	120	77	110	110	1	0	0	0	0	23	8.70	0
1068	2	9	133	28	15.83	88	102	53	110	108	107	102	71	112	117	114	108	81	112	114	113	112	72	110	117	1	0	0	0	1	29	8.80	0
1069	1	9	131	27	15.73	108	117	82	121	123	124	121	89	127	129	129	121	90	131	126	127	119	86	121	131	0	0	0	0	0	23	10.80	0
1070	1	10	138	35	18.38	82	100	61	96	95	98	104	78	89	96	93	111	77	96	101	99	112	79	92	101	1	1	1	1	1	19	8.20	0
1072	1	9	133	30	16.96	87	121	64	107	104	107	121	77	107	112	117	125	76	105	112	117	125	76	110	117	0	0	0	0	1	30	8.70	0
1074	1	8	127	26	16.12	102	106	60	108	109	115	112	70	120	122	123	116	69	120	127	115	119	75	120	127	0	0	1	1	0	25	10.20	1
1076	1	9	139	30	15.53	88	100	64	114	108	105	106	65	107	108	107	105	71	106	107	108	116	71	109	114	0	0	0	0	0	26	8.80	0
1079	1	10	128	28	17.09	80	106	60	101	98	103	111	78	102	104	97	111	73	103	104	104	116	67	105	105	1	0	1	0	0	25	8.00	0
1080	1	8	125	25	16.00	97	96	44	112	108	114	105	67	106	106	105	108	67	107	105	107	108	69	108	114	0	0	0	0	0	17	9.70	0
1081	2	9	136	30	16.22	81	108	71	97	104	110	112	67	116	114	110	108	72	116	112	118	113	68	114	118	0	0	0	0	1	37	8.10	0
1082	2	10	140	34	17.35	85	116	74	96	104	107	126	87	106	104	105	110	80	107	107	115	129	82	111	115	1	0	0	0	0	30	8.50	0
1083	2	9	143	38	18.58	98	103	64	108	106	104	118	83	103	107	109	113	82	108	105	109	115	77	112	112	1	0	0	0	1	14	9.80	0
1084	2	9	139	32	16.56	93	114	67	111	97	94	117	76	100	107	103	121	80	103	106	99	118	81	105	111	0	0	0	0	0	18	9.30	0
1085	1	9	140	42	21.43	100	114	70	114	114	109	125	79	105	111	114	126	81	106	112	111	128	77	106	114	0	0	0	0	0	14	10.00	0
1086	2	9	141	46	23.14	99	125	71	113	112	107	139	67	107	116	114	140	78	108	111	115	136	68	112	115	1	0	0	0	0	16	9.90	0
1087	2	9	126	25	15.75	89	106	75	114	108	115	114	76	118	116	113	114	82	122	124	124	113	82	127	127	0	0	0	0	0	38	8.90	0
1096	1	7	116	20	14.86	100	101	59	120	113	117	109	69	116	112	111	112	72	117	114	109	108	66	117	120	1	0	1	1	0	20	10.00	0
1097	1	8	131	26	15.15	100	107	53	115	123	126	117	68	124	125	126	117	66	129	120	120	112	62	120	129	0	0	0	1	1	29	10.00	0
1098	2	7	120	30	20.83	88	118	49	102	96	108	125	50	103	102	110	128	68	104	106	106	114	34	106	110	1	0	0	0	1	22	8.80	0
1100	1	7	123	25	16.52	97	106	61	108	114	117	108	67	116	118	120	101	59	120	112	109	89	57	117	120	1	0	0	0	1	23	9.70	0
1145	1	8	140	32	16.33	78	88	62	95	87	88	102	69	89	92	94	99	70	95	86	96	97	64	88	96	1	0	0	0	0	18	7.80	0
1147	2	8	129	26	15.62	99	111	80	106	108	108	113	82	109	102	107	118	78	111	111	115	118	80	113	115	0	0	0	0	0	16	9.90	0
1151	2	8	133	25	14.13	86	106	60	96	97	105	113	71	104	105	99	112	65	104	105	103	105	70	105	105	0	0	0	1	1	19	8.60	0

Test group

code	sex	age	height	weight	BMI	supine HR	supine SBP	supine DBP	HR1	HR2	HR3	SBP3	DBP3	HR4	HR5	HR6	SBP6	DBP6	HR7	HR8	HR9	SBP9	DBP9	HR10	highest HR	car sick	family history	water intake	sleeping hours	school-indiced burden	HR increase	supine HR10	POTS
1154	1	9	147	30	13.88	101	118	74	118	117	119	123	89	116	120	121	123	89	124	123	125	125	91	128	128	0	0	0	0	1	27	10.10	0
1155	2	9	120	34	23.61	84	114	54	89	97	84	113	78	88	90	90	115	70	93	94	94	113	72	89	97	1	0	0	0	1	13	8.40	0
1156	1	9	150	42	18.67	90	113	4	101	110	102	129	75	103	107	105	130	75	108	109	108	128	73	105	110	0	0	1	0	1	20	9.00	0
1159	2	9	140	35	17.86	86	118	57	104	103	93	128	78	94	103	93	121	80	102	92	96	127	82	102	104	0	0	0	0	0	18	8.60	0
1160	1	10	146	35	16.42	78	106	70	106	102	103	112	78	106	107	102	115	77	109	102	108	110	70	102	109	0	0	0	0	1	31	7.80	0
1161	2	9	135	30	16.46	70	110	60	89	92	90	105	71	94	88	93	105	73	97	94	90	102	72	95	97	1	0	1	0	0	27	7.00	0
1162	2	9	135	25	13.72	96	105	72	112	117	109	116	76	105	108	105	109	73	116	106	113	109	76	109	117	1	0	1	0	0	21	9.60	0
1165	2	8	126	22	13.86	84	103	57	99	98	97	112	77	99	100	97	107	77	98	102	97	109	70	102	102	0	0	0	0	1	18	8.40	0
1166	1	7	130	32	18.93	108	127	52	114	111	107	137	53	117	114	115	135	0	119	117	122	142	1	119	122	0	0	1	0	1	14	10.80	0
1167	2	7	124	26	16.91	73	88	61	98	80	81	100	71	86	85	83	104	70	82	84	87	108	8	83	98	0	0	0	0	1	25	7.30	0
1171	1	8	129	27	16.22	98	102	69	116	115	115	110	71	117	110	110	113	75	113	11	118	108	73	116	118	1	0	0	0	0	20	9.80	0
1174	1	7	118	22	15.80	99	95	58	107	108	109	105	69	107	114	111	10	69	108	109	108	103	68	106	111	0	0	0	1	0	12	9.90	0
1184	1	9	134	32	17.82	80	112	67	87	92	92	117	75	91	92	94	114	70	90	94	93	116	75	94	94	1	0	0	0	1	14	8.00	0
1200	1	9	137	32	17.05	76	120	67	87	80	84	131	74	85	93	97	123	82	91	98	97	114	69	96	98	0	0	0	0	0	22	7.60	0
1359	2	9	132	29	16.64	87	120	63	94	103	102	125	85	110	100	104	120	78	110	104	97	116	77	101	110	1	1	0	0	1	23	8.70	0
1360	2	8	135	31	17.01	96	116	74	109	106	102	115	80	103	109	96	115	76	106	103	105	114	83	101	109	1	0	0	0	1	13	9.60	0
1361	2	8	126	27	17.01	78	86	57	105	104	102	101	69	105	101	98	101	76	105	107	112	99	66	109	112	0	0	0	0	1	34	7.80	0
1366	2	10	143	29	14.18	81	112	73	97	95	100	120	87	96	95	91	118	80	96	97	96	118	83	95	100	1	0	0	0	0	19	8.10	0
1383	1	9	138	38	19.95	84	116	64	97	106	108	124	68	102	105	105	120	70	102	104	98	110	63	102	108	0	0	0	1	1	24	8.40	0
1388	2	7	140	34	17.35	86	94	54	91	102	105	102	51	104	108	113	109	62	101	109	107	104	63	106	109	1	0	0	0	1	23	8.60	0
1389	2	7	146	45	21.11	103	105	74	100	108	105	112	78	114	110	108	124	80	107	110	104	125	87	105	114	0	0	0	0	1	11	10.30	0
1390	2	7	133	31	17.53	80	107	62	96	95	92	113	66	99	96	99	109	74	94	95	91	111	73	94	99	0	0	0	0	0	19	8.00	0
1410	1	8	128	24	14.65	78	105	55	93	82	87	115	69	93	102	99	113	72	88	100	88	121	76	92	102	0	0	0	0	0	24	7.80	0
1411	2	7	118	21	15.08	107	96	61	109	109	110	112	68	116	118	119	115	76	117	119	128	114	67	125	128	0	0	0	1	1	21	10.70	0
1511	2	9	141	29	14.59	77	107	57	88	102	93	111	73	105	93	98	112	65	107	101	112	104	61	109	112	0	0	0	0	1	35	7.70	0
1512	1	9	138	40	21.00	92	117	58	102	106	111	114	75	111	110	109	117	76	112	112	110	122	67	111	112	0	0	0	0	0	20	9.20	0
1513	2	9	134	31	17.26	80	114	63	84	89	94	117	77	96	95	89	115	78	98	92	96	113	80	90	98	0	0	1	1	0	18	8.00	0
102A	2	12	148	56	25.57	88	113	69	104	101	94	118	76	98	98	97	113	80	101	91	97	121	76	94	104	1	0	0	0	1	16	8.80	0
102B	2	16	159	51	20.17	68	99	63	72	85	87	106	68	80	87	88	106	63	88	93	88	111	70	86	93	0	0	1	0	0	25	6.80	0
103A	1	12	151	42	18.42	80	111	70	94	87	90	123	85	90	103	92	126	75	91	95	98	120	76	95	103	0	0	0	0	1	23	8.00	0
103B	2	17	164	55	20.45	72	102	65	72	73	74	103	69	75	76	76	101	65	79	76	80	104	68	76	80	1	0	1	0	0	8	7.20	0
105A	2	13	158	44	17.63	96	114	63	88	88	96	123	65	100	103	96	119	67	94	97	99	120	78	96	103	1	0	1	0	0	7	9.60	0
105B	2	17	164	56	20.82	75	116	58	98	95	94	114	71	94	98	105	122	69	101	92	95	115	69	98	105	1	0	0	0	0	30	7.50	0
106A	1	12	156	40	16.44	85	110	72	100	95	90	119	79	98	90	91	116	78	90	102	89	117	78	91	102	1	1	1	0	1	17	8.50	0
106B	2	16	166	59	21.41	78	103	62	88	93	89	108	70	93	87	93	113	70	94	98	97	112	78	94	97	0	0	1	0	0	19	7.80	0
107A	2	12	155	43	17.90	94	105	68	93	92	97	107	78	94	97	108	107	75	98	110	110	108	73	105	110	0	0	1	0	1	16	9.40	0
107B	2	18	160	42	16.41	88	108	63	101	104	109	110	76	111	106	118	114	82	110	111	107	116	81	111	111	1	0	1	0	0	23	8.80	0
109A	1	13	101	50	49.01	81	111	65	98	103	97	123	87	100	92	97	121	80	99	102	99	121	79	101	103	1	1	1	0	1	22	8.10	0
109B	2	18	156	50	20.55	85	119	75	101	101	97	131	88	101	96	103	130	86	106	105	105	125	84	105	106	0	0	0	0	0	21	8.50	0
113A	1	12	157	49	19.88	87	102	66	96	94	103	113	82	105	104	100	113	77	103	105	104	110	79	103	105	1	0	1	0	1	18	8.70	0
113B	2	16	162	48	18.29	90	113	68	120	117	117	121	81	123	118	114	125	77	118	111	117	118	83	116	123	0	0	1	1	0	33	9.00	0
115A	2	11	157	42	17.04	75	112	58	90	93	88	116	80	100	99	98	112	70	94	101	98	116	74	103	103	1	1	1	0	1	28	7.50	0
115B	2	18	159	71	28.08	82	122	69	113	108	110	123	74	102	106	102	120	59	101	99	102	124	67	99	113	1	0	0	0	0	31	8.20	0
118A	2	12	145	38	18.07	85	100	55	83	85	86	103	64	87	95	95	104	66	94	95	93	105	70	93	95	0	0	0	0	1	10	8.50	0
118B	2	17	162	52	19.81	69	115	68	72	78	81	117	77	80	81	92	113	77	87	78	95	121	83	88	92	1	0	1	1	0	23	6.90	0
120A	2	12	159	44	17.40	78	124	72	90	88	92	135	93	91	101	103	133	91	92	110	106	133	95	100	110	0	1	0	0	0	32	7.80	0
120B	2	14	153	54	23.07	76	110	72	82	84	87	120	84	92	98	91	115	82	94	91	94	114	80	94	98	1	0	0	0	0	22	7.60	0
123A	2	14	154	56	23.61	86	115	67	89	85	84	128	78	90	92	87	125	77	81	89	95	118	73	89	95	1	0	0	0	0	9	8.60	0
123B	2	16	166	57	20.69	71	112	62	87	97	95	111	65	104	95	101	111	68	92	88	93	111	64	92	104	0	0	0	0	0	33	7.10	0
126A	2	12	158	44	17.63	73	119	66	76	74	78	120	78	79	88	75	125	85	84	78	82	120	81	84	88	1	0	0	0	0	15	7.30	0
126B	2	14	165	64	23.51	75	117	84	80	81	79	128	89	81	86	80	125	91	85	82	83	124	82	86	86	0	0	1	0	0	11	7.50	0
130A	2	12	148	42	19.17	86	105	66	95	92	92	105	73	93	92	94	108	71	100	100	94	106	72	97	100	1	0	0	0	1	14	8.60	0
130B	2	17	167	47	16.85	92	116	70	103	102	104	124	77	110	103	100	111	74	100	102	102	116	80	100	104	1	0	1	1	0	12	9.20	0
136A	2	13	158	50	20.03	100	110	69	115	120	118	122	83	114	120	121	120	84	125	122	118	119	83	117	125	1	1	1	0	0	25	10.00	1
136B	2	16	153	46	19.65	77	113	70	98	102	107	123	83	106	103	95	124	81	105	101	103	121	82	100	107	1	0	1	0	0	30	7.70	0

Test group

code	sex	age	height	weight	BMI	supine HR	supine SBP	supine DBP	HR1	HR2	HR3	SBP3	DBP3	HR4	HR5	HR6	SBP6	DBP6	HR7	HR8	HR9	SBP9	DBP9	HR10	highest HR	car sick	family history	water intake	sleeping hours	school-indiced burden	HR increase	supine HR10	POTS
138A	2	12	155	45	18.73	86	119	76	115	113	104	124	79	124	124	116	125	87	122	128	130	118	83	124	130	0	0	0	0	1	44	8.60	1
138B	2	17	159	69	27.29	72	120	74	64	64	66	125	76	79	74	74	122	72	74	73	73	118	72	72	79	1	0	1	0	0	7	7.20	0
140A	1	14	172	51	17.24	72	107	56	111	102	106	121	74	101	104	104	120	74	103	103	103	117	77	104	111	0	1	1	0	0	39	7.20	0
140B	2	17	161	51	19.68	74	113	71	86	87	89	121	82	87	87	86	123	84	86	83	79	122	80	83	89	1	0	1	1	0	15	7.40	0
150A	1	14	169	54	18.91	98	123	63	108	106	106	128	78	108	104	103	129	72	109	112	107	125	70	105	112	0	1	1	0	1	14	9.80	0
150B	2	17	159	61	24.13	60	100	63	64	65	71	107	67	70	70	69	101	66	71	69	68	103	64	72	70	1	0	1	0	0	10	6.00	0
152A	2	13	153	55	23.50	97	107	67	123	113	118	116	80	117	120	124	118	81	116	123	118	119	76	120	124	1	0	1	0	0	27	9.70	0
152B	2	16	158	53	21.23	67	106	71	59	73	78	108	69	78	84	77	108	70	81	91	80	104	68	81	91	1	0	1	0	0	24	6.70	0
155A	1	11	142	32	15.87	84	108	57	93	84	93	115	71	87	93	85	115	68	93	84	91	114	66	84	93	1	0	1	1	1	9	8.40	0
155B	2	15	161	54	20.83	75	113	62	90	98	101	112	72	100	97	102	119	66	102	104	102	121	72	100	104	1	0	0	0	0	29	7.50	0
156A	1	14	156	65	26.71	92	117	68	92	95	97	122	77	101	101	94	121	76	100	101	102	126	68	101	102	1	0	0	0	1	10	9.20	0
156B	2	17	158	55	22.03	78	122	62	82	90	91	122	73	94	90	92	124	68	93	93	92	121	72	93	94	1	0	0	0	0	16	7.80	0
157A	1	13	160	46	17.97	85	111	65	88	98	92	124	83	101	96	102	119	77	92	100	102	123	77	104	104	1	0	0	0	0	19	8.50	0
157B	2	14	162	60	22.86	79	113	61	106	79	90	122	70	85	82	80	118	68	86	99	87	121	60	90	106	1	0	0	0	1	27	7.90	0
158A	2	13	143	36	17.60	81	101	65	81	88	88	114	79	94	94	90	110	75	95	87	87	112	74	92	95	1	1	0	1	1	14	8.10	0
158B	2	18	165	60	22.04	74	122	57	74	74	76	117	61	74	78	74	120	52	78	75	75	113	64	75	78	1	0	0	0	1	4	7.40	0
159A	2	13	159	44	17.40	82	114	65	95	98	97	126	78	95	97	102	117	81	96	100	97	127	74	96	102	0	0	0	0	0	20	8.20	0
159B	2	18	158	53	21.23	67	107	67	78	85	83	111	74	90	86	87	109	72	89	87	91	110	73	89	91	0	0	0	1	0	24	6.70	0
160A	1	12	155	43	17.90	85	114	72	97	101	104	128	77	110	104	107	124	81	108	108	110	131	86	112	112	0	0	1	0	1	27	8.50	0
160B	2	16	160	64	25.00	80	118	56	81	80	85	119	71	96	90	88	122	76	87	82	86	122	69	88	96	1	0	0	0	0	16	8.00	0
161A	2	12	156	36	14.79	76	100	52	110	98	110	106	74	103	101	109	107	75	103	99	104	104	72	101	110	0	0	0	0	0	34	7.60	0
161B	2	18	162	60	22.86	85	126	82	82	80	90	124	81	94	96	95	129	81	98	97	101	131	91	95	101	0	1	0	0	0	16	8.50	0
162A	2	12	155	45	18.73	74	112	67	90	91	87	120	79	90	94	93	112	77	96	92	92	118	75	94	96	1	0	1	0	0	22	7.40	0
162B	2	17	158	64	25.64	60	121	75	67	70	71	123	77	74	76	75	118	75	80	72	80	121	73	76	80	0	0	1	0	0	20	6.00	0
163A	1	13	158	38	15.22	105	125	74	113	109	106	141	90	113	119	115	131	78	115	121		132	88		119	0	1	0	0	1	14	10.50	0
163B	2	16	163	45	16.94	75	109	75	78	73	81	117	81	73	75	78	110	72	75	76	77	114	83	76	81	1	0	1	1	1	6	7.50	0
167A	2	13	161	43	16.59	90	97	59	111	112	112	109	75	122	115	113	111	74	125	120	115	127	57	113	125	0	1	0	1	1	35	9.00	1
167B	2	16	158	40	16.02	61	100	64	71	71	73	108	72	70	69	72	108	68	72	75	78	108	67	76	78	0	0	1	1	0	17	6.10	0
174A	2	15	163	48	18.07	60	117	71	102	100	105	127	85	103	110	112	124	82	110	106	110	116	83	110	112	0	0	1	1	1	52	6.00	1
174B	2	17	159	50	19.78	78	104	71	94	89	87	114	81	91	88	86	113	80	92	88	92	107	75	94	94	1	0	1	0	0	16	7.80	0
180A	2	13	146	55	25.80	82	120	72	78	79	79	127	90	80	89	84	137	83	78	84	86	123	87	79	89	1	1	0	0	1	7	8.20	0
180B	1	15	177	70	22.34	85	122	79	117	113	113	125	85	122	118	109	138	81	116	117	115	125	88	122	122	0	0	1	0	0	37	8.50	0
181A	2	13	151	45	19.74	77	109	72	90	89	84	118	89	90	93	95	118	77	85	92	96	113	82	95	96	1	1	1	0	1	19	7.70	0
181B	1	17	178	61	19.25	93	104	63	116	117	120	112	69	119	121	124	114	74	112	118	122	115	69		124	1	0	0	0	0	31	9.30	0
182A	1	14	160	49	18.95	77	114	51	88	90	89	115	55	100	93	89	115	49	93	88	100	113	49	100	100	1	0	0	0	1	23	7.70	0
182B	1	17	167	67	24.02	93	118	65	100	101	100	122	77	102	104	104	125	75	102	103	104	126	77	103	104	0	0	1	1	0	11	9.30	0
183A	2	13	157	51	20.69	98	124	81	104	99	96	122	90	102	108	101	123	85	102	104	106	127	84	105	106	0	0	1	0	1	8	9.80	0
183B	1	13	158	41	16.42	78	107	64				118	77												77	0	0	0	0	1	-1	7.80	0
187A	1	12	151	41	17.98	77	105	54	82	86	92	116	72	92	91	93	110	60	94	99	100	105	67	96	100	1	1	1	1	1	23	7.70	0
187B	1	14	164	54	20.08	69	104	55	85	95	84	116	62	93	84	94	112	67	88	85	92	99	78	89	95	0	0	1	1	0	26	6.90	0
194A	2	13	165	50	18.37	82	117	66	88	105	98	125	72	107	105	100	130	79	107	111	108	124	75	112	112	1	1	0	0	1	30	8.20	0
194B	1	18	171	54	18.47	68	112	73	86	95	88	134	89	88	86	74	124	95	88	81	89	127	89	80	95	0	0	0	0	0	27	6.80	0
253A	2	14	163	56	21.08	75	109	69	98	89	76	143	93	74	86	90	123	87	91	90	91	127	84	92	98	1	1	1	1	1	23	7.50	0
253B	1	18	177	49	15.64	81	115	71	105	85	89	119	70	87	85	88	113	66	88	83	93	108	72	90	105	0	0	1	0	0	24	8.10	0
70A	1	12	149	37	16.67	91	106	64	105	106	108	116	82	109	110	112	114	73	109	116	117	115	77	118	118	1	0	0	0	0	27	9.10	0
70B	2	14	163	52	19.57	66	109	70	79	90	80	122	91	86	77	70	114	77	75	81	74	118	74	83	90	1	0	1	0	1	24	6.60	0
71A	1	12	167	45	16.14	103	107	70	92	86	92	130	85	90	92	92	121	84	88	90	95	124	84	86	95	0	0	1	0	1	-8	10.30	0
71B	2	16	160	48	18.75	85	106	64	122	112	112	118	68	121	124	120	112	71	114	121	113	114	67	112	124	1	0	1	0	0	39	8.50	0
75A	2	15	166	44	15.97	78	105	65	93	91	93	112	75	95	93	90	109	71	95	90	95	109	68	91	95	1	0	1	0	0	17	7.80	0
75B	2	16	154	82	34.58	82	110	72	83	88	88	119	85	93	94	88	122	88	91	94	82	123	83	85	94	1	0	1	1	1	12	8.20	0
