# Supplementary material for: Risk Factors for Postural Tachycardia Syndrome in Children and Adolescents
Source: PLoS One. 2014 Dec 4;9(12):e113625. doi: 10.1371/journal.pone.0113625 (PMC4256207; doi:10.1371/journal.pone.0113625)
Supplement: Table S2 — Data of the validation group. HR1 to HR10 recorded the steady heart rate in each min during the up-right position in the head-up test and 3 standing BPs at 3 minutes, 6 minutes and 9 minutes, respectively. The “highest HR” means the highest HR during the up-right position. “HR increase” means the highest HR minus supine HR. “supine HR 10” means “supine HR” divided by 10. Car sick: “1” stands for “yes” and “0” stands for “no”. Family history: “1” stands for “yes” and “0” stands for “no”. Water intake: “1” stands for less than 800 ml/day and 0 stands for more than 800 ml/day. Sleeping hours: “1” stands for less than 8 h/day and “0” stands for more than 8 h/day. School-induced burden: “1” stands for “yes” and 0 stands for “no”. POTS: “1” stands for patient and “0” stands for non-patient. Sex: “1” stands for male and “2” stands for female. BMI: body mass index = weight(kg)/height(m)2. (PDF) [file pone.0113625.s002.pdf]

Validation group

code	sex	age	height	weight	BMI	supine HR	supine SBP	supine DBP	HR1	HR2	HR3	SBP3	DBP3	HR4	HR5	HR6	SBP6	DBP6	HR7	HR8	HR9	SBP9	DBP9	HR10	Highest HR	car sick	family history	water intake	sleeping hours	school-induced burden	HR increase	supine HR10	POTS
3	1	12	156	55	22.60	83	107	62	86	95	95	117	75	95	90	93	111	72	90	89	88	114	70	92	95	1	0	0	0	1	12	8.30	0
8	1	12	163	70	26.35	75	135	77	78	77	83	142	89	93	84	94	158	89	88	92	86	146	75	87	94	1	0	1	0	1	19	7.50	0
13	2	12	157	41	16.63	108	117	67	115	119	121	127	88	123	123	124	118	80	119	120	120	120	84	125	125	1	0	0	0	0	17	10.80	0
14	2	15	167	43	15.42	87	109	78	99	97	99	109	80	100	102	99	104	76	102	111	108	104	74	106	111	1	0	0	0	0	24	8.70	0
17	2	12	153	42	17.94	85	108	67	90	92	97	126	89	99	90	95	120	82	95	96	97	117	81	107	107	0	0	0	0	1	22	8.50	0
19	2	12	155	38	15.82	88	109	70	101	109	119	116	78	118	116	114	111	71	118	121	121	114	74	123	123	0	0	0	0	1	35	8.80	0
20	2	11	138	32	16.80	117	115	78	138	132	130	127	90	128	125	127	121	86	131	128	124	124	83	124	138	0	0	0	0	0	21	11.70	0
22	1	13	160	48	18.75	75	108	63	99	81	82	121	72	87	89	96	113	79	87	90	91	114	74	86	99	1	0	0	0	1	24	7.50	0
26	2	18	155	59	24.56	84	134	81	83	88	100	129	84	90	97	93	128	84	92	96	94	122	84	97	100	1	0	1	0	1	16	8.40	0
29	1	12	160	42	16.41	77	119	66	103	87	97	123	85	87	90	95	117	81	97	91	92	118	70	95	103	0	1	0	0	1	26	7.70	0
32B	2	12	152	38	16.36	91	117	79	105	98	95	131	90	97	97	97	125	87	99	110	98	130	88	99	110	1	1	1	0	0	19	9.10	0
35	1	13	160	50	19.53	86	104	74	90	94	92	119	80	98	98	98	118	85	92	95	98	115	84	96	98	0	0	1	0	1	12	8.60	0
36B	2	17	165	64	23.51	78	108	62	95	98	98	114	71	94	92	96	112	70	97	98	96	107	66	97	98	0	1	0	1	1	20	7.80	0
40B	1	13	154	45	18.97	87	118	69	88	92	98	121	75	95	90	90	118	72	91	95	93	115	74	100	100	1	1	1	0	0	13	8.70	0
41B	1	13	166	47	17.06	85	105	59	85	86	86	113	67	90	89	99	112	69	91	97	92	109	68	91	99	0	0	0	0	0	14	8.50	0
46	1	12	145	35	16.65	79	113	63	95	83	88	121	73	90	88	89	113	62	90	93	93	110	69	92	95	0	1	0	0	0	16	7.90	0
48B	1	12	145	35	16.65	88	126	78	95	99	98	104	77	96	108	104	116	83	109	107	103	126	73	108	109	0	1	1	0	0	21	8.80	0
51B	1	12	152	48	20.78	89	114	63	104	110	108	121	81	109	107	111	124	78	106	101	104	125	69	108	111	1	0	0	0	0	22	8.90	0
54B	2	17	158	48	19.23	87	108	66	104	110	102	112	83	103	101	107	128	82	108	110	112	125	84	110	112	1	0	0	0	0	25	8.70	0
56	1	12	157	38	15.42	99	124	85	108	112	111	138	87	115	118	117	135	78	112	117	115	124	81	123	123	0	0	1	0	0	24	9.90	0
60B	1	13	175	80	26.12	88	141	77	119	109	115	143	87	114	115	113	153	79	109	119	120	139	90	112	120	0	0	0	0	0	32	8.80	0
61	2	12	161	50	19.29	73	109	71	88	81	84	114	78	81	90	87	124	74	81	87	83	116	77	90	90	0	0	0	0	1	17	7.30	0
75	1	13	154	45	18.97	90	110	68	120	111	108	121	82	120	113	105	123	84	114	115	118	122	78	123	123	1	0	0	0	1	33	9.00	0
80	1	12	153	48	20.50	93	137	73	104	103	97	124	81	97	101	100	135	75	104	100	103	135	76	98	104	1	1	1	0	0	11	9.30	0
83B	2	11	140	30	15.31	115	109	70	123	122	127	114	81	130	125	128	116	82	123	131	126	112	78	123	131	1	1	1	0	1	16	11.50	0
85B	1	13	158	42	16.82	72	105	64	97	97	93	112	77	96	102	92	109	73	98	98	98	115	61	103	103	1	1	0	0	1	31	7.20	0
87B	2	12	142	32	15.87	82	109	69	98	92	98	115	74	97	98	102	109	69	99	97	95	106	74	94	102	1	0	0	0	1	20	8.20	0
88B	1	13	151	47	20.61	80	104	62	98	96	93	108	73	96	97	94	109	72	96	96	88	108	70	92	98	0	0	1	0	1	18	8.00	0
91B	1	13	164	51	18.96	73	104	55	88	90	92	119	77	93	88	89	120	80	93	95	98	126	79	92	98	1	0	1	0	1	25	7.30	0
94B	1	13	158	40	16.02	73	108	62	98	106	101	114	65	109	109	108	110	69	108	113	112	107	71	111	113	0	1	1	1	1	40	7.30	1
97B	2	12	156	47	19.31	85	100	68	98	96	88	119	86	88	98	92	134	85	93	93	100	126	89	98	100	0	0	0	0	1	15	8.50	0
98	1	13	150	31	13.78	80	98	66	105	90	95	104	78	98	99	96	115	81	101	93	98	110	74	101	105	1	0	1	0	1	25	8.00	0
100	2	13	145	42	19.98	65	112	64	85	78	92	117	81	93	80	90	109	80	93	90	88	117	73	103	103	1	1	0	0	1	38	6.50	0
101B	2	12	150	33	14.67	102	107	80	110	109	118	134	76	111	114	116	117	79	114	116	112	113	81	124	124	1	0	0	0	1	22	10.20	0
108B	1	13	161	54	20.83	79	120	74	92	97	95	136	87	95	93	89	131	80	97	102	94	129	82	93	102	0	1	1	1	1	23	7.90	0
110B	1	13	157	42	17.04	72	99	68	79	80	78	104	76	79	78	77	107	74	88	90	87	109	76	85	90	1	1	1	0	0	18	7.20	0
114B	1	13	165	59	21.67	86	112	66	103	110	109	119	80	104	100	109	120	77	105	102	104	120	75	98	110	1	0	0	0	0	24	8.60	0
117B	1	14	144	35	16.88	72	110	66	86	90	97	111	75	93	93	97	109	74	95	95	94	114	73	93	97	0	0	0	0	1	25	7.20	0
124B	1	13	161	46	17.75	82	127	77	108	102	93	124	74	100	101	95	123	75	104	112	112	126	65	116	116	0	0	0	0	0	34	8.20	0
128B	1	13	158	49	19.63	78	119	65	74	81	81	117	74	88	85	84	122	68	79	91	85	119	70	89	91	0	0	0	0	1	13	7.80	0
129B	2	11	153	52	22.21	94	121	71	115	106	102	122	77	103	109	108	119	82	109	106	107	121	72	109	115	0	0	1	0	1	21	9.40	0
139B	1	13	162	40	15.24	75	97	62	95	100	95	106	75	98	100	97	113	82	101	97	98	111	79	100	101	0	0	0	1	1	26	7.50	0
147	1	13	149	36	16.22	95	115	71	118	106	112	119	77	111	113	114	114	78	113	113	114	119	75	120	120	0	0	1	0	1	25	9.50	0
165B	1	14	166	50	18.14	82	115	66	93	98	95	110	68	97	90	89	109	59	95	102	105	110	66	100	105	1	1	0	0	0	23	8.20	0
170B	2	14	156	48	19.72	103	107	66	113	101	103	120	84	115	116	117	118	87	103	108	106	125	88	107	117	1	0	1	0	1	14	10.30	0
173B	2	16	167	78	27.97	56	119	75	69	74	75	123	82	83	77	74	122	80	79	75	75	124	83	76	83	1	0	0	1	0	27	5.60	0
176B	2	16	157	41	16.63	65	110	72	79	92	78	122	87	86	87	82	113	82	78	84	88	114	82	82	92	0	0	1	1	0	27	6.50	0
183	1	17	170	56	19.38	81	123	89	98	95	89	128	88	92	90	94	124	87	95	90	100	122	79	92	100	0	0	0	0	0	19	8.10	0
184	1	16	166	65	23.59	77	125	75	108	99	103	128	78	103	103	103	120	74	100	103	96	115	75	103	108	0	0	1	0	0	31	7.70	0
186	1	16	166	55	19.96	72	113	68	83	87	83	118	75	85	103	93	117	69	96	90	96	115	69	90	103	0	0	1	1	0	31	7.20	0
188	1	13	148	37	16.89	76	99	58	93	91	91	99	68	97	98	98	106	70	91	97	97	103	58	99	99	1	0	0	0	0	23	7.60	0
189	1	17	166	50	18.14	75	111	72	90	94	90	124	85	87	93	89	116	81	88	99	93	119	82	85	99	0	1	0	1	0	24	7.50	0
190B	1	14	161	44	16.97	89	125	84	101	104	103	126	87	102	110	110	128	80	107	105	117	127	81	116	117	0	1	0	0	0	28	8.90	0
193	1	15	171	55	18.81	66	110	63	105	87	84	112	75	76	76	84	114	67	81	80	90	106	60	92	105	0	0	0	1	0	39	6.60	0
214	1	14	173	68	22.72	78	129	69	94	96	98	137	84	97	94	100	136	79	98	100	99	140	83	97	100	1	0	1	0	1	22	7.80	0

Validation group

code	sex	age	height	weight	BMI	supine HR	supine SBP	supine DBP	HR1	HR2	HR3	SBP3	DBP3	HR4	HR5	HR6	SBP6	DBP6	HR7	HR8	HR9	SBP9	DBP9	HR10	Highest HR	car sick	family history	water intake	sleeping hours	school-induced burden	HR increase	supine HR10	POTS
216	1	14	160	54	21.09	104	137	87	145	144	138	145	93	142	142	138	136	97	142	137	135	142	94	125	145	1	0	0	0	1	41	10.40	1
222	1	14	175	65	21.22	74	112	51	92	88	92	125	76	89	88	89	120	75	88	90	91	123	68	95	95	1	0	0	0	1	21	7.40	0
234	2	14	163	58	21.83	75	124	66	96	91	95	148	85	91	92	100	140	85	95	94	90	136	94	87	100	1	0	0	0	1	25	7.50	0
238	2	14	165	50	18.37	87	124	77	95	92	97	130	80	102	97	104	126	74	98	103	103	132	78	103	104	0	1	1	0	1	17	8.70	0
258	1	18	164	61	22.68	63	105	51	75	84	86	110	75	82	83	83	104	70	84	78	80	113	75	80	86	0	0	0	0	1	23	6.30	0
261	1	18	176	61	19.69	75	104	69	91	94	84	115	86	92	89	88	109	84	86	84	80	111	79	86	94	1	0	1	0	0	19	7.50	0
482	1	11	149	50	22.52	90	113	68	112	113	110	122	75	115	113		127	67	114	114	120	124	75	119	120	0	0	1	1	1	30	9.00	1
500	1	11	166	54	19.60	63	97	55	90	87	92	98	57	90	97	94	99	63	101	109	108	102	60	112	112	1	0	0	0	1	49	6.30	1
503	1	10	152	39	16.88	104	133	83	121	125	120	140	97	128	122	118	153	90	124	125	119	142	89	127	128	1	0	1	1	0	24	10.40	1
555	1	11	158	46	18.43	108	124	72	122	120	115	123	82	126	124	119	124	80	131	126	111	124	76	114	131	1	0	0	0	1	23	10.80	1
701	2	11	160	52	20.31	88	115	60	91	90	98	119	76	96	97	95	124	72	94	94	95	122	64	95	98	1	0	0	0	0	10	8.80	0
702	2	11	147	56	25.92	88	109	66	90	93	101	117	75	102	102	110	117	59	105	109	108	118	63	109	110	1	0	0	0	1	22	8.80	0
703	1	11	152	36	15.58	77	102	54	90	97	96	107	69	94	92	91	111	65	98	100	96	118	70	94	100	0	0	0	0	1	23	7.70	0
706	1	11	154	53	22.35	92	114	60	96	103	101	120	75	97	102	102	114	76	105	106	104	119	68	105	106	1	1	0	0	1	14	9.20	0
708	2	10	139	30	15.53	65	94	53	90	75	86	101	70	86	84	81	99	67	85	84	87	106	65	84	90	0	0	0	0	1	25	6.50	0
709	2	11	149	40	18.02	96	119	61	120	105	104	123	83	105	105	105	114	75	109	107	105	117	57	106	120	0	0	0	0	0	24	9.60	0
711	1	11	151	60	26.31	64	122	61	79	75	88	130	86	87	89	88	133	71	83	86	85	119	69	84	89	0	0	0	1	1	25	6.40	0
712	2	11	141	27	13.58	75	95	44	84	95	94	103	58	98	94	99	104	60	98	94	96	100	62	104	104	1	1	0	0	1	29	7.50	0
713	1	11	151	39	17.10	76	109	72	84	82	90	129	85	95	93	96	126	83	97	95	94	119	82	95	97	0	0	0	1	0	21	7.60	0
714	1	11	142	42	20.83	95	100	51	104	109	112	113	75	108	110	105	114	83	108	105	108	113	78	107	112	1	0	0	0	0	17	9.50	0
715	2	10	140	32	16.33	93	104	63	103	97	96	123	75	93	101	106	112	71	102	105	103	118	69	104	106	1	0	0	0	1	13	9.30	0
716	2	10	126	23	14.49	90	85	46	94	100	106	120	76	108	104	103	113	83	100	101	102	121	77	101	108	1	1	1	1	1	18	9.00	0
717	1	11	139	42	21.74	83	117	54	106	107	104	125	67	106	103	108	125	65	104	108	112	125	71	108	112	0	0	0	0	1	29	8.30	0
719	1	10	142	40	19.84	92	125	79	94	97	95	135	84	96	102	94	131	74	94	98	93	124	82	92	102	1	0	0	0	1	10	9.20	0
720	2	10	141	32	16.10	95	105	60	97	102	104	112	80	103	105	106	114	81	105	100	105	113	76	105	106	1	1	0	0	1	11	9.50	0
721	1	11	139	33	17.08	78	96	60	96	104	104	106	73	109	104	103	114	76	104	105	107	111	79	105	109	0	0	0	0	0	31	7.80	0
723	1	10	149	38	17.12	78	105	49	104	104	96	122	77	102	103	104	118	77	100	103	106	119	66	102	104	0	0	0	0	0	26	7.80	0
724	1	11	147	49	22.68	71	103	63	108	103	100	115	80	102	102	94	117	79	103	91	97	124	85	95	108	0	0	0	1	1	37	7.10	0
729	1	11	151	45	19.74	84	103	62	93	97	91	106	73	96	95	96	107	77	91	94	95	106	72	95	97	1	0	0	0	1	13	8.40	0
732	2	10	128	26	15.87	71	102	53	81	90	94	112	64	88	90	93	107	68	90	93	91	107	59	85	94	1	0	0	0	1	23	7.10	0
733	2	10	137	29	15.45	83	100	54	88	102	98	105	67	101	86	94	102	63	96	91	95	108	67	98	102	0	0	0	0	0	19	8.30	0
734	1	10	141	40	20.12	74	99	50	102	98	101	114	71	102	101	100	112	68	100	102	102	109	67	101	102	1	0	0	0	0	28	7.40	0
735	1	10	142	37	18.35	82	112	62	88	94	104	126	79	99	101	98	118	91	96	97	99	129	72	95	104	1	0	0	0	1	22	8.20	0
736	1	11	140	43	21.94	88	94	58	98	106	100	104	71	110	103	103	107	65	102	103	103	104	73	103	110	0	0	0	1	0	22	8.80	0
737	2	10	143	41	20.05	100	127	75	116	113	110	146	81	110	116	108	146	88	110	111	106	137	81	104	116	1	0	0	0	1	16	10.00	0
740	2	11	147	41	18.97	81	109	51	100	95	98	113	68	92	95	94	108	64	98	95	89	106	64	90	100	1	0	0	1	1	19	8.10	0
742	1	11	142	33	16.37	97	114	64	110	108	108	125	74	106	112	108	123	79	113	115	110	123	73	109	115	1	0	0	0	1	18	9.70	0
743	1	11	144	32	15.43	78	110	51	87	95	90	111	71	94	100	93	108	73	96	90	87	112	69	87	100	0	0	0	0	0	22	7.80	0
744	1	11	139	34	17.60	75	123	70	84	85	88	117	73	90	95	93	115	76	96	96	95	118	74	97	97	1	0	0	0	1	22	7.50	0
747	2	11	143	33	16.14	98	107	65	100	86	90	122	73	91	92	94	112	82	94	93	92	111	77	95	100	1	0	0	0	0	2	9.80	0
748	1	11	148	45	20.54	85	97	63	109	95	99	116	79	97	95	98	122	66	95	93	98	100	68	95	109	0	0	0	0	0	24	8.50	0
749	1	11	145	46	21.88	90	94	64	111	118	10	115	67	112	108	113	110	64	112	109	108	111	77	111	118	1	0	1	0	1	28	9.00	0
752	2	11	141	33	16.60	82	108	58	92	103	108	109	61	96	98	101	109	64	97	109	107	110	71	107	109	1	1	0	0	1	27	8.20	0
753	1	11	152	51	22.07	100	115	73	110	108	110	127	87	106	113	108	127	89	116	116	107	130	84	112	116	1	1	0	0	0	16	10.00	0
759	2	11	155	49	20.40	74	117	68	108	101	102	126	81	104	99	96	124	81	101	95	99	119	83	98	108	0	0	0	0	1	34	7.40	0
760	2	11	150	34	15.11	76	111	55	103	95	94	117	74	101	98	99	119	67	99	97	102	116	65	103	103	0	0	0	0	0	27	7.60	0
761	2	10	137	29	15.45	90	103	48	97	98	100	113	59	105	105	105	105	44	104	108	105	103	56	109	109	1	0	0	0	1	19	9.00	0
764	2	10	130	26	15.38	113	107	69	123	123	113	119	81	114	110	126	119	77	117	118	119	116	74	115	126	1	1	0	1	1	13	11.30	0
765	2	10	136	38	20.54	80	110	51	87	96	91	113	67	97	105	97	126	61	94	93	92	120	58	94	105	1	0	0	1	0	25	8.00	0
767	2	11	147	36	16.66	105	124	71	116	117	117	138	79	119	127	120	131	82	123	125	128	130	84	125	128	0	0	0	0	0	23	10.50	0
768	2	10	146	37	17.36	77	114	54	85	105	88	113	66	89	90	98	115	67	90	90	88	109	67	87	105	1	0	0	1	1	28	7.70	0
769	1	10	130	29	17.16	95	112	67	102	101	99	117	84	101	101	102	119	80	102	98	96	118	82	98	102	0	0	0	0	0	7	9.50	0
773	2	10	146	45	21.11	85	107	45	105	105	105	120	64	102	105	100	119	64	105	102	101	111	74	104	105	0	0	0	0	0	20	8.50	0
774	1	11	145	43	20.45	92	107	70	106	96	104	118	82	106	106	104	125	72	109	102	105	117	76	104	109	0	0	0	0	0	17	9.20	0
775	2	10	139	30	15.53	111	100	63	118	121	120	103	74	119	127	119	110	79	126	131	133	115	85	123	133	1	0	0	0	0	22	11.10	0

Validation group

code	sex	age	height	weight	BMI	supine HR	supine SBP	supine DBP	HR1	HR2	HR3	SBP3	DBP3	HR4	HR5	HR6	SBP6	DBP6	HR7	HR8	HR9	SBP9	DBP9	HR10	Highest HR	car sick	family history	water intake	sleeping hours	school-induced burden	HR increase	supine HR10	POTS
777	1	10	144	35	16.88	81	101	63	98	113	107	124	85	108	103	102	115	74	102	103	101	111	81	106	113	1	0	0	0	1	32	8.10	0
778	1	11	143	35	17.12	86	105	71	122	109	118	126	83	117	118	114	124	89	119	117	119	127	86	115	122	0	0	1	1	1	36	8.60	1
779	1	11	134	26	14.48	113	115	65	122	127	123	120	86	122	126	121	123	78	120	123	125	125	83	122	127	1	1	1	1	0	14	11.30	1
784	1	11	142	39	19.34	90	122	57	101	92	95	122	85	95	93	96	124	77	95	94	97	132	72	95	101	1	0	0	1	0	11	9.00	0
786	2	8	122	33	22.17	87	111	48	90	93	95	104	83	104	103	104	105	58	97	96	98	102	52	98	104	1	0	0	0	1	17	8.70	0
787	1	8	133	26	14.70	104	106	58	118	116	121	125	76	122	121	122	114	54	122	124	116	109	67	117	124	0	0	0	0	0	20	10.40	0
791	1	8	148	45	20.54	80	127	62	102	96	97	128	68	100	99	102	116	72	100	101	98	127	66	105	105	0	0	0	0	1	25	8.00	0
795	2	8	126	23	14.49	82	103	59	84	86	91	108	74	92	94	95	106	74	96	96	94	112	73	93	96	0	0	0	0	0	14	8.20	0
797	1	10	130	28	16.57	89	109	77	105	103	104	128	93	98	105	108	136	96	103	107	102	134	92	105	108	0	1	0	0	0	19	8.90	0
803	1	8	125	25	16.00	82	74	29	85	87	89	105	62	90	95	92	107	72	88	90	89	103	58	92	95	0	0	0	0	0	13	8.20	0
804	1	8	144	25	12.06	84	103	68	113	106	111	107	72	112	114	113	112	73	109	114	109	110	76	123	123	1	0	0	0	1	39	8.40	0
805	1	10	148	30	13.70	85	119	72	105	103	103	134	77	105	105	103	132	78	104	108	105	132	85	103	108	0	0	0	0	0	23	8.50	0
806	1	10	140	33	16.84	86	88	69	104	103	99	100	84	104	105	103	106	72	105	104	106	107	71	99	106	0	0	0	0	0	20	8.60	0
808	2	11	149	38	17.12	84	108	52	101	97	92	119	72	94	95	93	121	71	96	98	95	118	69	95	101	1	0	0	0	1	17	8.40	0
809	2	9	137	31	16.52	78	105	60	94	97	101	111	66	104	103	100	110	68	103	97	99	106	71	108	108	1	0	1	0	0	30	7.80	0
810	2	9	141	30	15.09	97	106	72	136	134	128	119	82	133	126	130	123	81	131	134	135	108	76	134	136	0	0	0	0	0	39	9.70	0
811	2	10	137	24	12.79	72	91	60	77	81	85	112	68	90	88	87	97	59	81	86	84	100	73	87	90	0	1	0	0	1	18	7.20	0
812	1	10	134	28	15.59	76	113	59	98	82	91	105	60	92	90	92	113	66	92	93	95	113	61	95	98	1	1	0	0	1	22	7.60	0
813	2	9	140	30	15.31	97	106	67	109	99	100	114	80	104	106	101	104	77	97	105	101	105	73	100	109	0	0	0	0	1	12	9.70	0
817	2	9	146	37	17.36	110	108	58	121	114	120	113	67	125	117	125	117	67	122	123	122	111	68	119	125	1	0	1	0	0	15	11.00	0
818	1	9	146	27	12.67	113	109	65	109	111	110	115	82	114	118	108	117	76	114	116	113	119	76	116	118	0	0	0	0	1	5	11.30	0
819	2	10	137	30	15.98	90	102	59	103	107	95	104	45	104	109	102	103	62	103	110	107	90	65	114	114	0	0	0	0	0	24	9.00	0
821	1	10	144	33	15.91	84	112	75	110	101	98	123	85	106	98	102	120	84	109	104	101	122	65	100	110	1	0	0	1	1	26	8.40	0
822	1	10	148	56	25.57	102	117	72	114	120	119	119	83	111	116	116	117	64	116	116	114	123	85	117	120	1	0	1	1	0	18	10.20	1
824	1	10	141	33	16.60	89	128	57	114	108	112	132	76	115	112	119	120	78	118	122	117	118	81	111	122	1	0	0	0	0	33	8.90	0
825	2	10	148	40	18.26	90	106	62	94	97	100	115	79	107	105	101	131	79	105	97	100	129	64	97	107	1	1	0	0	1	17	9.00	0
833	1	10	145	50	23.78	103	101	57	120	119	117	110	73	119	117	104	108	71	118	110	115	115	73	118	120	1	0	0	0	1	17	10.30	0
834	2	10	136	33	17.84	97	109	59	111	105	112	124	75	109	110	107	119	76	109	105	103	108	70	105	112	1	0	0	0	1	15	9.70	0
836	2	9	137	34	18.11	86	111	71	110	106	106	125	88	112	117	113	117	78	114	117	117	118	80	118	118	1	1	1	0	1	32	8.60	0
839	2	10	147	30	13.88	80	118	77	93	97	91	116	82	97	96	97	114	77	98	95	100	114	78	90	100	0	0	0	1	0	20	8.00	0
840	2	9	152	28	12.12	89	107	80	122	117	113	121	76	115	114	122	124	85	120	118	114	123	83	118	122	0	0	0	0	0	33	8.90	0
842	1	10	137	30	15.98	84	100	66	93	94	93	106	74	92	103	94	101	66	97	103	99	102	65	100	103	1	1	1	0	1	19	8.40	0
844	2	8	145	22	10.46	101	107	74	117	112	110	118	72	126	115	117	107	77	120	113	121	117	76	130	130	0	0	0	0	1	29	10.10	0
845	2	9	150	39	17.33	87	105	67	92	85	97	112	74	93	95	92	109	70	98	97	93	110	75	94	98	0	1	0	0	1	11	8.70	0
847	2	8	143	21	10.27	88	100	68	116	103	106	104	75	108	103	101	109	75	109	107	100	105	73	113	116	1	0	0	0	1	28	8.80	0
848	1	10	147	40	18.51	96	114	55	109	111	108	125	76	104	107	107	120	80	109	114	108	122	74	108	114	1	0	0	0	1	18	9.60	0
849	2	10	127	28	17.36	75	111	70	88	86	89	110	83	96	92	90	106	78	91	95	96	115	83	97	97	1	1	0	0	1	22	7.50	0
851	2	10	142	46	22.81	107	131	89	141	129	136	127	98	126	128	126	135	89	134	130	126	129	79	128	141	1	0	1	1	0	34	10.70	1
854	1	9	136	28	15.14	82	101	42	104	100	101	108	63	107	109	109	109	64	111	107	112	105	67	111	112	0	0	0	0	0	30	8.20	0
864	1	8	129	32	19.23	76	104	71	113	114	109	114	78	115	112	108	108	70	121	117	112	109	72	117	121	0	0	1	1	1	45	7.60	1
874	1	10	134	34	18.94	70	101	58	96	98	94	106	65	89	100	103	106	63	103	101	94	105	26	78	103	0	1	0	1	1	33	7.00	0
876	1	10	135	30	16.46	74	93	64	102	95	104	103	65	92	90	94	99	66	95	90	97	108	54	90	104	0	0	0	0	0	30	7.40	0
878	1	10	132	28	16.07	75	97	51	97	95	85	114	72	87	89	91	92	70	88	91	87	134	41	90	97	1	1	0	0	0	22	7.50	0
881	1	10	142	39	19.34	76	103	58	87	87	87	99	62	89	92	88	102	63	93	93	91	96	67	96	96	1	0	0	0	1	20	7.60	0
882	1	10	149	50	22.52	75	110	50	96	90	86	127	100	93	97	90	122	76	90	96	98	121	73	95	98	0	0	0	1	1	23	7.50	0
885	2	10	132	25	14.35	90	110	67	106	95	103	115	78	102	101	105	119	75	106	106	109	116	76	107	109	1	0	0	0	0	19	9.00	0
893	1	9	130	25	14.79	84	110	57	82	95	104	89	62	104	100	105	84	52	97	96	94	101	65	95	105	1	0	0	0	0	21	8.40	0
897	1	9	134	34	18.94	100	108	61	108	105	108	121	69	108	105	110	115	72	106	107	109	120	71	109	110	1	0	0	0	0	10	10.00	0
899	2	10	133	31	17.53	95	125	81	124	123	121	88	69	123	125	127	137	89	128	123	128	137	96	128	128	1	0	0	0	0	33	9.50	0
904	1	10	146	33	15.48	120	132	76	125	123	125	134	86	123	125	121	136	83	125	123	125	131	84	119	125	1	1	0	0	1	5	12.00	0
905	1	10	143	34	16.63	92	97	55	105	105	98	105	73	104	110	106	106	69	105	107	105	107	73	104	110	1	0	0	1	1	18	9.20	0
910	1	9	136	48	25.95	98	120	63	112	110	110	125	74	110	112	114	118	76	109	114	111	120	75	109	114	0	0	0	0	0	16	9.80	0
912	1	10	134	28	15.59	84	105	67	101	99	102	110	72	105	102	110	108	72	106	108	105	113	70	108	110	1	1	0	1	0	26	8.40	0
913	1	10	153	50	21.36	105	120	75	113	110	114	135	90	114	112	112	134	88	117	116	117	125	82	114	117	1	0	0	0	0	12	10.50	0

Validation group

code	sex	age	height	weight	BMI	supine HR	supine SBP	supine DBP	HR1	HR2	HR3	SBP3	DBP3	HR4	HR5	HR6	SBP6	DBP6	HR7	HR8	HR9	SBP9	DBP9	HR10	Highest HR	car sick	family history	water intake	sleeping hours	school-induced burden	HR increase	supine HR10	POTS
916	2	9	139	49	25.36	82	104	58	104	107	106	103	71	104	107	101	97	60	102	105	107	100	66	107	107	1	1	1	0	1	25	8.20	0
920	1	9	137	30	15.98	80	103	45	106	109	104	108	72	103	94	94	110	72	93	108	107	108	73	103	109	0	0	0	0	0	29	8.00	0
929	1	7	132	25	14.35	98	109	71	119	124	119	117	83	121	123	119	118	86	125	118	125	114	81	123	125	1	0	0	0	0	27	9.80	0
940	2	7	128	23	14.04	94	117	73	107	102	113	126	82	103	106	96	117	79	98	108	105	119	74	110	113	1	0	0	0	0	19	9.40	0
944	1	11	142	34	16.86	108	119	77	113	116	113	131	94	118	120	117	132	91	112	114	117	125	89	117	120	1	0	0	0	1	12	10.80	1
946	1	10	141	36	18.11	89	116	78	106	106	109	132	91	107	117	116	129	88	124	108	107	126	91	118	124	1	1	0	0	0	35	8.90	0
947	1	10	142	51	25.29	78	101	48	94	94	100	117	76	85	93	100	115	68	96	90	100	112	70	100	100	0	0	0	1	0	22	7.80	0
948	2	10	142	33	16.37	87	133	73	95	95	97	101	53	96	94	96	100	77	99	95	96	102	59	97	99	1	1	0	0	1	12	8.70	0
949	2	10	136	36	19.46	100	103	63	110	110	107	106	69	108	108	115	109	69	111	114	112	108	70	113	115	1	0	0	0	0	15	10.00	0
950	2	10	135	30	16.46	97	100	60	108	112	122	111	82	120	125	122	107	53	125	115	123	102	73	125	125	0	0	0	0	0	28	9.70	0
951	2	10	140	37	18.88	108	115	67	116	117	117	119	66	121	118	117	126	44	111	116	116	105	67	118	121	1	1	0	1	1	13	10.80	0
952	2	10	143	30	14.67	107	109	76	122	114	117	116	87	113	118	117	116	80	113	118	115	110	78	120	122	1	1	0	1	1	15	10.70	0
953	2	10	148	41	18.72	91	108	36	105	106	111	115	50	110	111	113	122	49	112	110	113	114	49	109	113	1	0	0	0	1	22	9.10	0
963	2	8	138	32	16.80	82	94	55	112	115	113	109	70	116	116	122	115	81	126	129	131	123	84	128	131	0	0	1	1	1	49	8.20	1
1013	1	7	140	30	15.31	82	106	65	116	118	122	112	75	110	112	112	114	79	118	125	123	112	79	112	125	0	0	1	1	1	43	8.20	1
1028	2	10	148	34	15.52	80	104	67	92	105	102	105	72	104	102	99	108	74	99	102	109	115	74	108	109	1	1	0	0	1	29	8.00	0
1092	1	7	126	24	15.12	88	102	62	103	105	103	112	72	100	103	107	107	71	102	99	98	108	68	97	107	0	0	0	0	1	19	8.80	0
1093	2	7	125	26	16.64	86	96	60	111	114	113	101	65	116	112	113	99	70	118	120	123	109	74	122	123	1	0	1	1	1	37	8.60	0
1094	2	7	125	24	15.36	95	105	70	123	127	128	89	66	126	125	129	110	70	130	132	127	125	76	127	132	1	0	0	0	1	37	9.50	0
1255	1	7	126	25	15.75	85	115	52	96	102	101	114	65	98	100	105	113	64	103	105	100	117	67	105	105	0	0	0	0	0	20	8.50	0
1363	2	9	133	26	14.70	109	102	68	128	131	133	110	81	126	136	135	115	79	129	133	133	111	75	137	137	1	0	1	1	0	28	10.90	1
177A	2	14	155	46	19.15	83	95	56	98	98	98	111	66	102	99	103	107	71	94	88	102	104	71	102	103	1	0	1	0	1	20	8.30	0
177B	2	16	169	67	23.46	64	121	76	77	71	79	127	85	81	90	81	124	82	87	89	85	121	77	81	90	1	0	1	1	0	26	6.40	0
192A	1	14	172	49	16.56	86	104	58	110	104	106	173	150	109	108	109	116	74	105	103	108	116	69	108	110	0	0	1	0	1	24	8.60	0
192B	1	17	179	62	19.35	76	124	69	91	86	83	139	86	83	93	87	132	76	96	88	89	136	67	85	96	0	0	0	0	0	20	7.60	0
195A	1	14	164	53	19.71	86	109	59	105	114	103	123	73	110	109	107	123	72	94	107	105	111	78	106	114	1	0	1	0	0	28	8.60	0
195B	1	16	166	56	20.32	88	125	77	100	92	89	134	86	91	87	86	125	80	98	95	94	128	80	98	100	0	0	1	0	0	12	8.80	0
1A	1	13	148	38	17.35	96	115	67	99	107	100	127	78	106	102	102	120	79	104	106	114	125	88	108	114	1	0	1	0	1	18	9.60	0
1B	2	16	169	65	22.76	80	105	67	85	80	77	127	81	79	81	82	116	74	85	86	82	109	67	86	86	1	0	0	0	1	6	8.00	0
44A	1	14	166	51	18.51	85	109	67	102	102	104	115	81	100	107	101	112	77	98	99	102	115	81	99	107	0	1	1	0	0	22	8.50	0
44B	2	15	157	49	19.88	72	110	67	86	86	88	118	71	86	83	85	111	69	85	82	85	106	69	83	88	1	0	1	0	1	16	7.20	0
50A	1	11	158	41	16.42	68	111	63	75	78	81	117	75	88	78	80	117	78	83	84	78	116	77	80	88	0	0	1	0	0	20	6.80	0
50B	1	12	143	33	16.14	81	95	61	78	80	95	110	76	91	85	87	114	81	88	90	88	115	78	85	95	1	0	1	0	0	14	8.10	0
